# Neuroimaging Paradigms to Identify Patients for Reperfusion Therapy in Stroke of Unknown Onset

**DOI:** 10.3389/fneur.2018.00327

**Published:** 2018-05-15

**Authors:** Mark R. Etherton, Andrew D. Barreto, Lee H. Schwamm, Ona Wu

**Affiliations:** ^1^Department of Neurology, JPK Stroke Research Center, Massachusetts General Hospital (MGH), Harvard Medical School, Boston, MA, United States; ^2^Stroke Division, Department of Neurology, McGovern Medical School at the University of Texas Health Science Center at Houston, Houston, TX, United States; ^3^Department of Radiology, Athinoula A. Martinos Center for Biomedical Imaging, Massachusetts General Hospital (MGH), Charlestown, MA, United States

**Keywords:** ischemic stroke, neuroimaging, reperfusion therapy, unwitnessed stroke, wake-up stroke

## Abstract

Despite the proven efficacy of intravenous alteplase or endovascular thrombectomy for the treatment of patients with acute ischemic stroke, only a minority receive these treatments. This low treatment rate is due in large part to delay in hospital arrival or uncertainty as to the exact time of onset of ischemic stroke, which renders patients outside the current guideline-recommended window of eligibility for receiving these therapeutics. However, recent pivotal clinical trials of late-window thrombectomy now force us to rethink the value of a simplistic chronological formulation that “time is brain.” We must recognize a more nuanced concept that the rate of tissue death as a function of time is not invariant, that still salvageable tissue at risk of infarction may be present up to 24 h after last-known well, and that those patients may strongly benefit from reperfusion. Multiple studies have sought to address this clinical dilemma using neuroimaging methods to identify a radiographic time-stamp of stroke onset or evidence of salvageable ischemic tissue and thereby increase the number of patients eligible for reperfusion therapies. In this review, we provide a critical analysis of the current state of neuroimaging techniques to select patients with unwitnessed stroke for revascularization therapies and speculate on the future direction of this clinically relevant area of stroke research.

## Introduction

The treatment options for acute ischemic stroke are currently predicated on a confirmed last-known well (LKW) and the time-period from LKW to hospital evaluation. For those patients that present and start treatment within 4.5 h from LKW, administration of intravenous recombinant tissue plasminogen activator (IV tPA) reduces disability after acute ischemic stroke (1, 2). Likewise, those with large-vessel occlusions (LVO) of the anterior circulation who can start treatment within 6 h of LKW, endovascular thrombectomy (EVT) is a powerful therapy for improving long-term functional outcomes (3–6). Recently, two pivotal trials (7, 8) now extend that window up to 24 h in highly selected patients with imaging demonstrating small infarct core lesions and salvageable tissue by imaging or clinical measures. Unfortunately, these efficacious acute therapies are limited both by the relatively narrow treatment window for either IV tPA or EVT, and the relatively infrequent (5.7–12.8%) occurrence of LVO accompanied by a favorable tissue signature in the later time windows (9). Because of this, many ischemic stroke patients are not eligible for these therapies. While the rates of thrombolysis are increasing in the United States, conservative estimates in 2009 suggested that only 3–5% of all stroke patients receive treatment with IV tPA (10). One reason for its underuse may be the strict time restrictions from LKW (11). Exacerbating this issue, current estimates suggest that 31–36% of acute ischemic stroke patients have stroke of unknown symptom onset (SUSO) but do have an LKW time (12, 13), with a large proportion of these with deficits upon awakening, or “wake-up strokes” (WUS) (14–16). These patients with SUSO highlight the challenge of relying on a human witness of symptom onset, which greatly limits the opportunities for reperfusion therapy.

**Figure 1:**
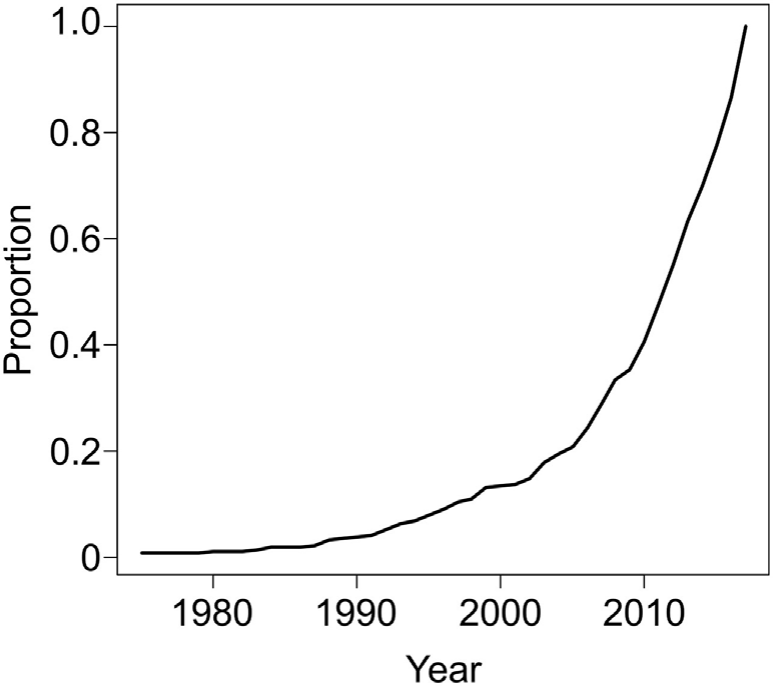
PUBMED search on January 11, 2018 (294 hits, 112 relevant) using the following terms: “stroke"[Title/Abstract] AND ("unwitnessed"[Title/Abstract] OR "unclear onset"[Title/Abstract] OR "unclear-onset"[Title/Abstract] OR "wake"[Title/Abstract] OR "wakeup"[Title/Abstract] OR awake* [Title/Abstract] OR "unknown onset"[Title/Abstract] OR "unknown-onset"[Title/Abstract]) AND ("trial"[Title/Abstract] OR therap* [Title/Abstract] OR treat*[Title/Abstract] OR thrombolysis [Title/Abstract]) NOT ("review"[Publication Type] OR "review literature as topic"[MeSH Terms]) NOT ("animals"[MeSH Terms:noexp] OR animal[All Fields]) demonstrate increasing interest in the treatment of patients with unknown symptom onset restricted up to December 31, 2017.

For these reasons, there is much interest in developing novel approaches to expand patient eligibility for revascularization therapies (e.g., IV tPA or EVT) to SUSO patients. Given the potentially large proportion of ischemic stroke patients that these populations represent, identifying approaches to discern which patients with SUSO may still safely benefit from reperfusion therapy holds enormous clinical and epidemiological ramifications. The interest in this question is exemplified by the increasing number of publications on this topic (Figure 1). Advanced neuroimaging has been applied to patients with SUSO based on two principles: (1) to substitute for the human witness of stroke onset by providing radiographic surrogates for stroke duration or (2) to identify patients with sufficient salvageable tissue at risk of dying to make the potential benefit of revascularization therapy worth the risk and considerable resource utilization of “late” intervention. The DAWN (DWI or CTP Assessment with Clinical Mismatch in the Triage of Wake-Up and Late Presenting Strokes Undergoing Neurointervention with Trevo) and DEFUSE 3 (Endovascular Therapy Following Imaging Evaluation for Ischemic Stroke) randomized clinical trials (RCTs) used different approaches to identify these potential candidates for EVT, and their success has much to teach us about the patients who can still benefit from late-window reperfusion. In this review, we will discuss the current evidence supporting the use of neuroimaging approaches for evaluation of patients with SUSO to identify populations that may benefit from delayed reperfusion interventions.

## Wake-Up Stroke as a Distinct Class of SUSO

Wake-up strokes are hypothesized to represent a unique entity within SUSO as it is difficult to delineate the timing of stroke onset including the possibility that it may have occurred on awakening (17). Many have posited that there is a circadian variation in the frequency of ischemic stroke with most strokes occurring between 6:00 AM and 12:00 PM (15, 18–22). A meta-analysis of 8,250 patients with ischemic stroke demonstrated a 55% increased risk between 6 AM and noon (22). The diurnal variation in ischemic stroke is also thought to have contributions from morning increases in blood pressure, platelet aggregation, and prothrombotic factors (23–25). These observations have led many to speculate on circadian-related mechanisms underlying WUS, similar to those reported for myocardial infarction, and that WUS patients may have stroke onset contiguous with wakening. Multiple studies have in fact shown comparable presentation and outcomes in WUS patients vs. those with witnessed stroke onset (14, 19, 26, 27). One large study that investigated the cohort of WUS patients enrolled in the International Stroke Trial found that WUS patients, despite presenting with milder symptoms, had similar mortality rates and likelihood of poor outcome as patients with stroke onset while awake (28).

On the other hand, non-wake-up SUSO patients appear to represent a different clinical population than WUS SUSO patients. One study demonstrated that non-wake-up SUSO patients differ clinically from wake-up SUSO patients (more severe symptoms, faster arrival time from symptom discovery). Non-wake-up SUSO patients also appear to have worse prognosis than either WUS or witnessed stroke patients and the proportion of patients with non-wake-up SUSO may be increasing (29, 30). These findings suggest that both wake-up and non-wake-up SUSO patients represent a vulnerable subpopulation of AIS patients in need of developing new management paradigms for expanding reperfusion therapy eligibility. With the success of the late-window EVT trials (7, 8) and new guideline recommendations (11), the clinical focus should shift to expanding therapies for late-window patients without LVO or who lack rapid access to advanced neuroimaging and EVT.

## SUSO vs. Stroke of Known Symptom Onset (SKSO)

### Computed Tomography (CT)

There have been several CT-based approaches to characterizing SUSO patients as compared with their witnessed stroke counterparts. Multiple studies have demonstrated no significant difference in the extent of ischemic changes on the admission CT between WUS SUSO and SKSO patients (31–33) using the Alberta stroke program early CT score (ASPECTS) scale, which is a CT-based assessment of early ischemic changes in the middle cerebral artery (MCA) territory (34). Further supporting the argument that WUS SUSO patients may represent a distinct population, a study comparing cardioembolic SKSO (46 patients), non-WUS SUSO (18 patients), and WUS (17 patients) observed no significant difference between the SKSO and WUS groups in the number of patients presenting with a normal head CT (30 vs. 22%, *P* = 0.76) or hypodense area (0 vs. 11%, *P* = 0.069) (26). However, no patients in the non-WUS SUSO group had a normal head CT and 56% had a visualized hypodense area (*P* < 0.001) (26). Another study used CT perfusion (CTP) to characterize 420 stroke patients with known symptom onset, 131 patients with WUS, and 125 with non-wake-up SUSO (35). The non-wake-up SUSO group had larger lesion volumes on CT-angiogram source images compared with the other two groups (46.6-cm^3^ SUSO vs. 14.3-cm^3^ SKSO vs. 14.4-cm^3^ WUS, *P* = 0.04) but no difference in the frequency of CTP mismatch or presence of LVO (35).

### Magnetic Resonance Imaging (MRI)

Magnetic resonance imaging-based approaches to identifying WUS patients who may benefit from reperfusion have also been performed. A retrospective study of 364 stroke patients, which included 100 patients with WUS, showed no differences in median stroke severity, as assessed by National Institutes of Health Stroke Scale (NIHSS) score (SKSO 7 vs. WUS 5; *P* = 0.06), age, or gender between the WUS and known stroke onset groups (14). Notably, while time from stroke onset was shorter in the known stroke onset group (6.0 vs. 13.3 h, *P* < 0.001), there was no significant difference in time from symptom detection (6.0 vs. 5.9 h, *P* = 0.83) (14). Of those patients imaged within 3 h of symptom discovery (*N* = 69), there was no difference in either the diffusion-weighted imaging (DWI: 26.8-cm^3^ SKSO vs. 19.6-cm^3^ WUS) or perfusion-weighted imaging (PWI) lesion volumes (107.7-cm^3^ SKSO vs. 82.7-cm^3^ WUS) (14).

Imaging findings of non-WUS SUSO patients have also been characterized with MRI, though not to the same extent as the WUS cohort. One retrospective study found that non-WUS SUSO patients (*N* = 104) were more likely to have DWI and FLAIR (fluid-attenuated inversion recovery) mismatch (non-WUS SUSO: 35.1 vs. WUS: 21.9%; *P* = 0.02), and DWI-PWI mismatch (*P* = 0.001) than WUS (*N* = 172) (13). However, a prospective study of SUSO patients found that the frequency of DWI-FLAIR mismatch (DFM), defined as a visible acute lesion on DWI but no obvious parenchymal hyperintensity in the corresponding region of the FLAIR sequence, was similar in both the WUS and non-WUS SUSO groups (43.7 vs. 48.7%; *P* = 0.3) (30).

## Neuroimaging Time-Stamp of Stroke Duration

**Figure 2:**
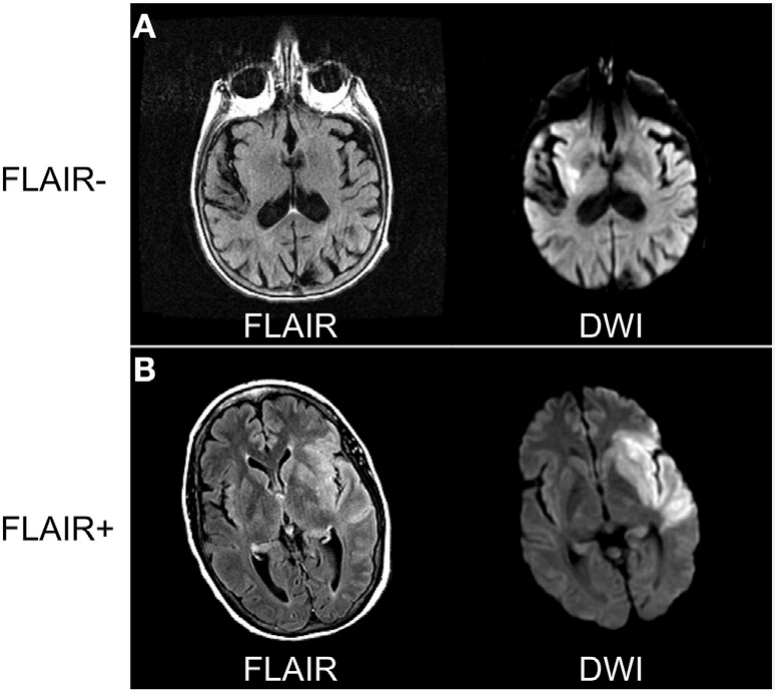
Comparing diffusion-weighted imaging (DWI) and FLAIR sequences to determine radiographic time of stroke onset. **(A)** Eighty-one-year-old woman awoke with dysarthria and right-sided weakness. Magnetic resonance imaging (MRI) performed 8 h from last-known well (LKW) shows signal abnormality in DWI but not FLAIR sequences. **(B)** Sixty-three-year-old woman developed sudden onset right-sided weakness with confirmed LKW. MRI performed 5 h from LKW shows signal abnormality on both DWI and FLAIR sequences consistent with stroke onset > 4.5 h. Data analysis for figure was created under approval of local ethics committee.

The aforementioned studies suggest that SUSO patients present with similar clinical and imaging findings as their SKSO counterparts, as long as they are evaluated within a comparable time frame from stroke onset. This observation has prompted the utilization of imaging as a potential surrogate witness of onset when no human witness is available. Before using an imaging-based witness, it is critical to determine the key imaging features that can discriminate between patients within the therapeutic time window and those who are outside the window. Imaging-surrogates for stroke duration have been primarily based on MRI, specifically, the DWI (or apparent diffusion coefficient, ADC) and FLAIR sequences. Several clinical studies of acute ischemic stroke patients have strengthened the argument that patients with abnormal ADC and normal FLAIR are likely within 3–4 h of stroke onset and that assessing for DFM may be associated with stroke duration (Figure 2) (36–39).

In one study of AIS patients with brain MRI obtained within 12 h of stroke onset, the median time from known symptom onset was significantly longer in FLAIR-positive vs. FLAIR-negative patients (189 min, interquartile range 110–369 vs. 103 min, interquartile range 75–183 min; *P* = 0.011) (39). Moreover, in patients with infarct volume exceeding 0.5-cm^3^ on DWI, FLAIR-negative MRI showed 80% specificity and 51% sensitivity for imaging within 3 h of stroke onset (39). However, the authors observed no significant correlation between the signal intensity ratio and time from stroke onset. In contrast, another study showed a strong positive correlation between the time from stroke onset and the intensity of the FLAIR signal change relative to its contralateral homologous region (40). These findings support the idea that with longer stroke duration, the likelihood of visible FLAIR abnormalities increases. This allows for the hypothesis that patients with visible changes on DWI (or ADC) but normal FLAIR will likely have relatively recent stroke onset.

Several studies have strengthened the idea that DFM can inform on stroke duration. One retrospective investigation of 120 patients with AIS within 6 h of known symptom onset showed that presence of DFM identified patients with stroke onsets within 3 h or less with 93% specificity and 48% sensitivity (36). Importantly, 98.3% of the study population had confirmed arterial occlusions. Those patients that were FLAIR-positive were imaged significantly later than the FLAIR-negative group (180 vs. 120 min, *P* < 0.001) (36). In a retrospective, multicenter follow-up to this study involving 543 patients with AIS, DFM identified with 78% specificity and 62% sensitivity patients within 4.5 h and 87% specificity and 56% sensitivity patients within 6 h from stroke onset (41). Two additional studies demonstrated that scans with DFM were highly specific (71–80%) for identifying patients within 3 h of stroke onset (37, 38). Lastly, another study demonstrated that the presence of DFM on 3T MRI also has a high positive predictive value (88%) for the stroke occurring within 4.5 h; however, 44.5% of this population had positive FLAIR within 4.5 h of stroke onset and would not considered DFM (42). These results suggest that on 3T MRI, the presence of DFM can identify patients with stroke onset <4.5 h with high specificity but that a significant percentage of patients in the <4.5 h window can have positive FLAIR signals. Taken together, these findings demonstrate that MRI can, with high specificity, identify patients in the hyperacute (i.e., <3–4.5 h) phases of AIS based on DFM.

Using neuroimaging to serve as a radiographic biomarker of stroke onset holds much promise for potentially expanding eligibility for thrombolytic therapy. One analysis of WUS patients with DFM suggested that an additional 30% would be eligible for treatment with IV tPA (43). As such, several clinical trials have asked the question of whether DFM can be safely and efficaciously used for the treatment of SUSO patients with thrombolytics.

## Moving Beyond the Clock: Shifting the Paradigm from “Time is Brain” to “Imaging is Brain”

Complementary to the notion that imaging can serve as a surrogate for stroke duration is that imaging can directly measure the degree of injury the brain has already experienced from the ischemic event. While the duration of time since symptom onset is highly correlated with progression of brain tissue injury, there is tremendous between-subject variability as to the rate of tissue death in the face of a heterogeneous degree of ischemia. While one can calculate an average rate of neuronal death in AIS due to LVO (1.9 million neurons/min) (44), recent data confirm that many patients still have viable tissue well beyond 6 h after symptom onset. Early animal models of ischemic stroke have supported the hypothesis that mismatch between the DWI and T2-weighted signals reflect histologically salvageable tissue, which happen to also be associated with short stroke durations (45). This has prompted different neuroimaging approaches to quantify or characterize salvageable tissue as a radiographic surrogate of patients likely to benefit from reperfusion therapies. The importance of using neuroimaging to identify patients likely to benefit from reperfusion therapies is exemplified, in part, by the results of the International Management of Stroke III RCT (46), which enrolled 53.4% of subjects with no baseline CTA to confirm LVO and 45% of subjects with ASPECTS <8, and failed to show benefit of EVT, as compared with the positive EVT trials of 2015, which used stricter criteria for identifying LVO patients with small ischemic cores (4, 5, 47, 48). However, this trial used mostly first- and second-generation devices and it is unknown what the impact would have been if stent retrievers had been used. In the next sections, we will review the different imaging-based approaches to quantify viable tissue in patients with AIS independent from LKW.

### Infarct Core–Perfusion Mismatch

One approach has been to apply MRI or CT-based imaging techniques to quantify infarct core–perfusion mismatch as an indicator of salvageable tissue. While these two modalities measure core in very different ways, they both seek to differentiate irreparably injured tissue from tissue that is potentially recoverable.

#### MRI-Based Perfusion–Diffusion Mismatch

**Figure 3:**
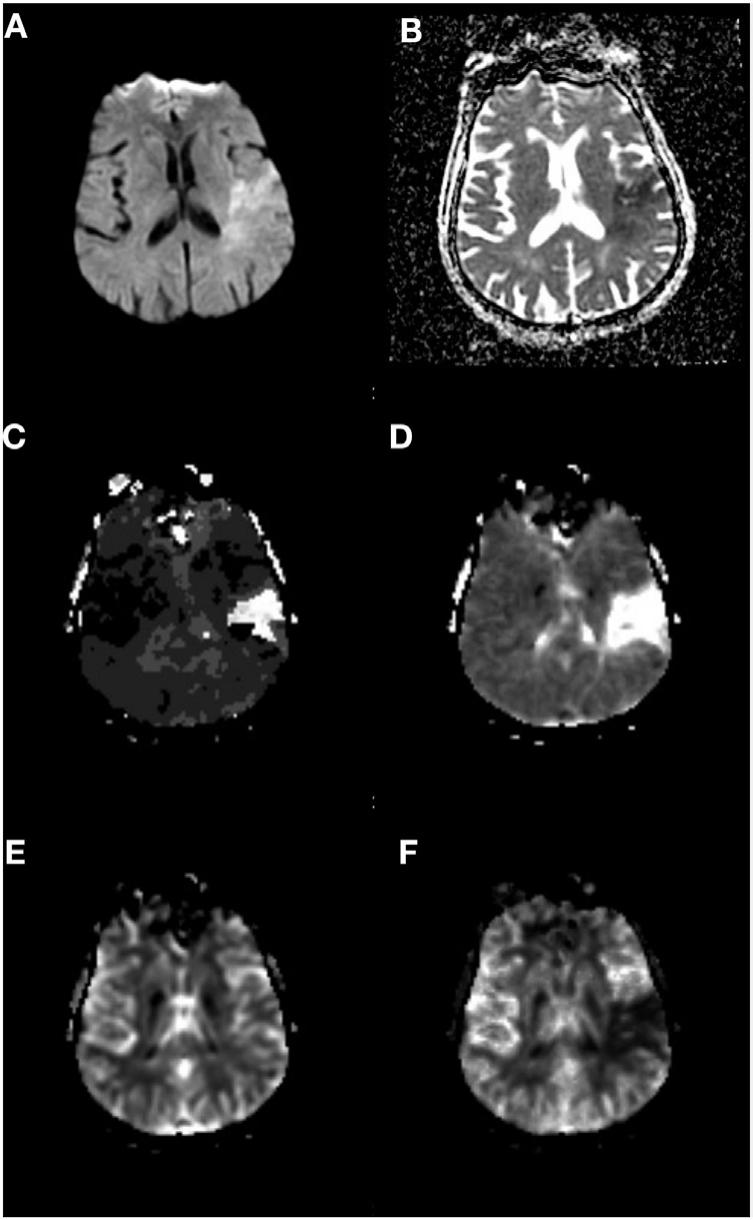
Perfusion–Diffusion mismatch to identify salvageable tissue. **(A)** Diffusion-weighted imaging and **(B)** apparent diffusion coefficient sequences showing mismatch of ischemic core to a greater volume of hypoperfused tissue on **(C)** *T*_max_ and **(D)** mean transit time sequences. **(E)** Cerebral blood volume and **(F)** cerebral blood flow sequences. Data analysis for figure was created under approval of local ethics committee.

Magnetic resonance imaging-based techniques are one method that has been utilized to quantify salvageable tissue. Tissue that is abnormal on DWI due to restricted diffusion typically represents tissue that has the highest probability of infarction, with tissue salvage rare even with reperfusion (49), and therefore typically referred to as the infarcted “core” (50). PWIs, in particular gadolinium-arrival time measures such as *T*_max_ [time to peak value of deconvolved residue function (51)], have been used to identify tissue that is at risk for ischemic infarction but has not yet irreversibly committed to cell death (45, 51–56). These observations represent the foundations of utilizing PWI–DWI mismatch to identify salvageable tissue as an alternative triage approach for SUSO patients (Figure 3).

Investigating this hypothesis, the Diffusion and Perfusion Imaging Evaluation for Understanding Stroke Evolution (DEFUSE) study was an observational study of IV tPA-treated patients for which target perfusion–diffusion mismatch was defined as mismatch volume (PWI–DWI) > 10 cm^3^ or mismatch ratio (PWI/DWI) > 1.2. PWI lesion was defined as tissue exhibiting *T*_max_ ≥ 2 s. MRI was obtained before and 3–6 h after IV tPA treatment. A total of 68% of the study population had a confirmed partial or complete arterial occlusion of the internal carotid artery (ICA), MCA, or posterior cerebral artery (PCA). DEFUSE enrolled 74 patients and found that 56% (*N* = 18) with perfusion–diffusion mismatch and early reperfusion had a favorable outcome (defined as improvement of NIHSS between baseline and 30 days of 8 points or more or score of 0–1 at day 30) (57).

In a subsequent prospective cohort study, DEFUSE 2 (58), the same approach was applied to patients with LVO of the anterior circulation (defined as intracranial ICA or first segment of the MCA) treated with EVT within 12 h of LKW. The target mismatch profile was notably modified from that used in DEFUSE; mismatch ratio > 1.8 (*T*_max_ > 6-s volume/DWI volume) and an absolute difference ≥15 cm^3^, DWI lesion volume <70 cm^3^, and *T*_max_ > 10-s volume <100 cm^3^. In the 78 patients with target mismatch, the adjusted odds ratio for favorable outcome (same definition as DEFUSE) with reperfusion was 8.8 (95% CI 2.7–29) compared with 0.2 (95% CI 0–1.6) in the no target mismatch group (*P* = 0.003). Moreover, in the target mismatch group, reperfusion was associated with decreased infarct growth at 5 days (30 vs. 73 cm^3^, *P* = 0.01). It should be noted that both DEFUSE and DEFUSE 2 were observational studies with treatment decisions made independent of imaging findings, which introduce selection bias; once enrolled, all patients received IV tPA or endovascular intervention.

#### CT-Based Infarct Core–Perfusion Mismatch

**Figure 4:**
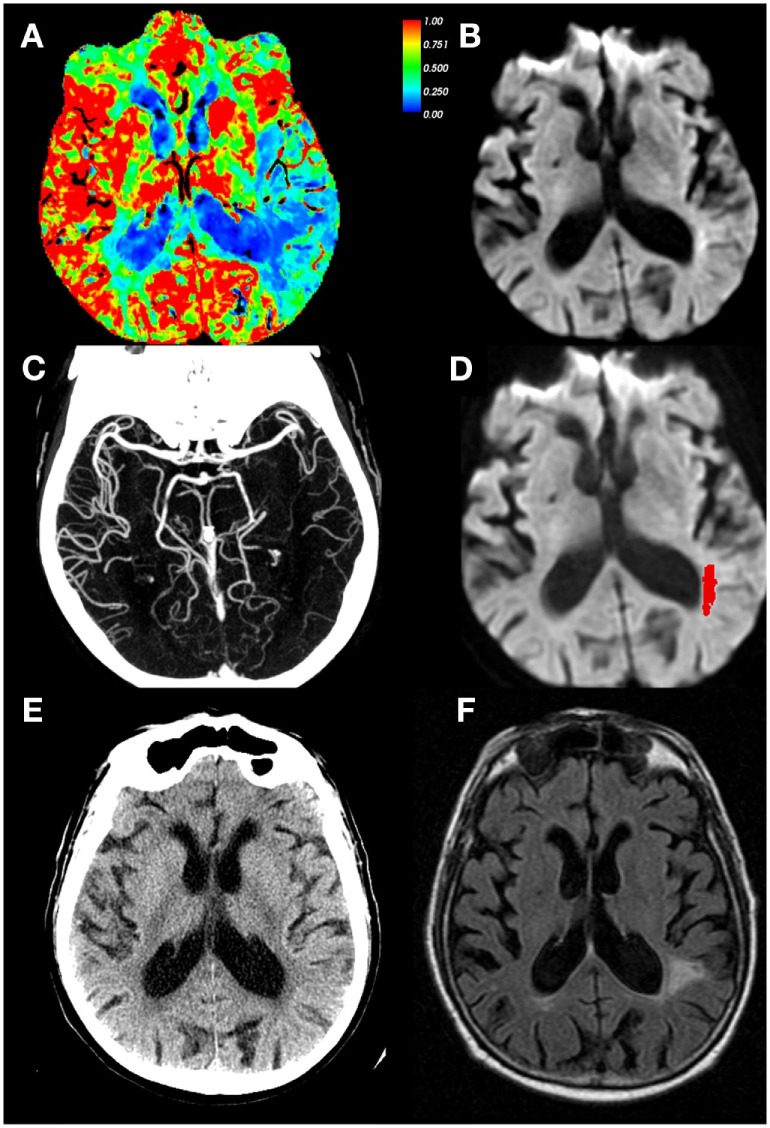
CT perfusion (CTP) cerebral blood flow (CBF) maps do not correspond with infarct core. Eighty-four-year-old male with left middle cerebral artery (MCA) stroke with dense distal M2 occlusion presenting with initial National Institutes of Health Stroke Scale (NIHSS) of 9. By the time of the admission, NIHSS was 2 and patient did not receive IV tPA or endovascular treatment. The CTA/ CT perfusion (CTP) was acquired at 4.9 h from last-known well (LKW), magnetic resonance imaging was performed 19 min after the CTP. **(A)** CBF, **(B)** diffusion-weighted imaging (DWI) performed 19 min after CTP, **(C)** CTA shows occlusion of MCA superior division segment, **(D)** DWI with acute infarct mapped in red, **(E)** CT head at 24 h from LKW, and **(F)** follow-up FLAIR image at 34 days post-stroke depicting final infarct. Note the CTP CBF hypoperfused region identified as 30% of mean contralateral hemisphere is much larger than that of the DWI scan, and corresponded better with tissue infarction on follow-up. *Courtesy of William A. Copen, MD, Department of Radiology, Massachusetts General Hospital. Data analysis for figure was created under approval of local ethics committee*.

Due to the relative insensitivity of non-contrast CT for detecting early ischemic changes (59), alternative methods of defining core lesion volume are needed for CT-based screening methods. Thresholded relative cerebral blood flow (rCBF) maps have been used to approximate core lesion volumes, though, unlike DWI, they do not measure tissue infarction (Figure 4). Rather they are based on the probabilistic association that areas with substantive hypoperfusion are highly likely to progress to infarction despite reperfusion. These estimates can be highly variable at two extreme conditions: (1) patients with LVO stroke imaged early at stroke onset exhibiting large rCBF lesion volumes that grossly overestimate final infarct volume in settings of early reperfusion, and (2) patients with many hours of occlusion imaged after late reperfusion demonstrating small or no CBF volumes (due to normal or increased CBF values in previously hypoperfused tissue) that would grossly underestimate final infarct volume. However, these conditions are infrequent, and many trials and centers have adopted a CT-based approach to identify subjects with tissue at risk, using very low values in CTP-derived CBF values to define “core,” with CTP-derived tracer arrival time metrics used to represent tissue at risk (5) at centers which do not perform acute stroke MRI. Although the accuracy of using a perfusion metric to define infarction “core” is still debated (60–62), this approach has successfully identified a group of patients who respond to reperfusion therapy beyond 3- and 4.5-h time windows (see below). Some have suggested using thresholded relative cerebral blood volume (rCBV) maps to identify core (63) or absolute CBV values <2 cm^3^/100 g (64, 65), but many studies have shown that CBV is not a robust surrogate for infarct core (66–71). However, investigators have shown that very low CBV might be an indicator of risk for future hemorrhagic transformation (72–75) or poor outcome after EVT (76, 77).

#### Clinical Trials of Infarct Core–Perfusion Mismatch Involving Non-SUSO Patients

There have been several trials applying the principles of infarct core–perfusion mismatch in the administration of IV thrombolytics to late-window AIS patients with varying degrees of success (Table 1). The Desmoteplase in Acute Ischemic Stroke Trials (DIAS and DIAS-2) used an alternative thrombolytic, desmoteplase (more specific for fibrin than alteplase) and core–perfusion mismatch (>20%) for the treatment of late-window AIS patients 3–9 h from LKW (78, 79). DIAS was a dose escalation study of desmoteplase. Phase 1 of DIAS was halted because of high rates of sICH with desmoteplase doses of 25–50 mg (26.7%) (78). Phase 2 of DIAS, however, showed that with desmoteplase doses of 62.5–125 µg/kg the rates of sICH were 2.2% and reperfusion rates were 71.4 vs. 19.2% with placebo. Of note, reperfusion in this trial was defined as a ≥30% reduction in mean transit time or ≥2 points improvement on the Thrombolysis in Myocardial infarction grading scale (78). Although the trial was not powered to detect efficacy, at 90 days there was a dose-dependent rate of favorable outcome (defined as Barthel index > 75, modified Rankin scale (mRS) ≤ 2, and NIHSS 0–1 or improvement of 8 points) of 60% with 125 µg/kg vs. 18.2% placebo (78). The Dose Escalation of Desmoteplase for Acute ischemic Stroke (DEDAS) trial was a placebo-controlled, dose-escalation study of 90 and 125-µg/kg desmoteplase in 37 patients 3–9 h from LKW (80). No sICH occurred in any group and there appeared to be a dose-dependent effect of desmoteplase on reperfusion rates (53.3% 125-µg/kg desmoteplase vs. 18.2% 90-µg/kg desmoteplase vs. 37.5% placebo) (80). In DIAS-2, 186 patients were randomized to either 90 or 125-µg/kg desmoteplase or placebo 3–9 h from LKW utilizing the same infarct core–perfusion mismatch criteria. Notably, in addition to MRI, CTP was also used in DIAS-2 for assessing infarct core–perfusion mismatch (64 patients); however, mismatch was determined based on a visual, qualitative assessment. DIAS-2 had a favorable safety profile but there was no difference in the rates of favorable outcome at 90 days, median change in infarct volume, or rates of sICH (79).

**Table 1:** Randomized clinical trials of delayed intravenous thrombolysis or EVT in acute ischemic stroke beyond 3 h.

Study	Study drug	Imaging selection	No. of treated	Time window	sICH definition	Rate of sICH (%)	Primary outcome: intervention vs. placebo
EPITHET (84)	Alteplase	MRI (PWI/DWI mismatch)	52	3–6 h	SITS-MOST	7.7	Infarct growth between baseline and 90 days. Median infarct growth ratio 0.66 (95%CI 0.36–0.92), *P* = 0.054.

DEDAS (80)	Desmoteplase	MRI (PWI/DWI mismatch)	29	3–9 h	ECASS II	0	Reperfusion at 4–8 h 18.2% (90 µg/kg), 53.3% (125 µg/kg) vs. 37.5% (placebo). Good clinical outcome^a^ 28.6% (90 µg/kg), 60% (125 µg/kg) vs. 25% (placebo)

DIAS part 2 (78)	Desmoteplase	MRI (PWI/DWI mismatch)	57	3–9 h	ECASS II	2.2	Reperfusion rates 71.4 vs. 19.2%. Favorable clinical outcome^a^ 13.3% (62.5 µg/kg), 60% (125 µg/kg) vs. 22.2% (placebo)

DIAS II (79)	Desmoteplase	MRI (PWI/DWI mismatch) or CTP	125	3–9 h	ECASS II	3.5–4.5	Favorable clinical outcome^a^ 47% (90 µg/kg), 36% (125 µg/kg), 46% (placebo)

DIAS 3 (82)	Desmoteplase 90 µg/kg	CTA/MRA high-grade stenosis or occlusion (<1/3 ACA/PCA or <1/2 MCA)	247	3–9 h	ECASS II	3	90-day mRS 0–2: 51% vs. 50% (aOR 1.2, 95%CI 0.79–1.81; *P* = 0.4).

DIAS 4 (83)	Desmoteplase	CTA/MRA high-grade stenosis or occlusion (<1/3 ACA/PCA or <1/2 MCA)	124	3–9 h	ECASS II	4.8	90-day mRS 0–2: 41.9% vs. 35.9% (OR 1.45, 95%CI 0.79–2.64; *P* = 0.23)

ECASS III (1)	Alteplase	CT (<1/3 MCA)	418	3–4.5 h	≥4pt ↑ NIHSS at 72 h due to ICH	2.4	90-day mRS 0–1: 52.4% vs. 45.2% (OR 1.34, 95%CI 1.02–1.76; *P* = 0.04).

EXTEND^c^ (121)	Alteplase	MRI (PWI/DWI mismatch) or CTP	400	3 or 4.5–9 h	SITS-MOST	NA	90-day mRS 0–1.

MR RESCUE (89)	EVT	MRI or CTP (voxel-based algorithm)	64	<8 h	SITS-MOST	4	Median 90-day mRS: 3.9 vs. 3.9.

EXTEND-IA (5)	EVT	CTP mismatch	35	4.5–6 h	SITS-MOST	0	Reperfusion at 24 h: 100% vs. 37% (aOR 27.0, 95%CI 5.5–135.0; *P* < 0.001). Early neurologic improvement^d^: 80% vs. 37% (aOR 6.0, 95%CI 2.0–18.0; *P* = 0.002)

SWIFT-PRIME (4)	EVT	MRI (PWI/DWI mismatch)	98^b^	<6 h	≥4pt ↑ NIHSS at 24 h due to ICH	0	90-day mRS 0–2: 60% vs. 35% (RR 1.70, 95%CI 1.23–2.33; *P* < 0.001)

ESCAPE (48)	EVT	Multiphase CTA and collateral status	120	<12 h	≥2pt ↑ NIHSS due to any ICH	3.6	90-day mRS 0–2: 53% vs. 29.3% (cOR 2.6, 95%CI 1.7–3.8; *P* < 0.001).

*^a^Combined analysis defined at 90 days as ≥8 point improvement or scoring 0 to 1 on NIHSS, score of 0 to 2 on mRS, and a BI score of 75 to 100*.

*^b^Eighty-three patients treated using PWI/DWI mismatch. Fifteen patients treated based small-core defined as ASPECTS ≥6 on CT or MRI*.

*^c^Trial completed or terminated but not yet published. Trial in progress*.

*^d^Early neurologic improvement defined as reduction of eight points or more on NIHSS or score of 0 or 1 at 72 h*.

*ACA, anterior cerebral artery; CTA, CT angiogram; CTP, CT perfusion; DWI, diffusion weighted imaging; EVT, endovascular thrombectomy; MCA, middle cerebral artery; mRS, modified Rankin scale; NIHSS, National Institutes of Health Stroke Scale; PCA, posterior cerebral artery; PWI, perfusion weighted imaging*.

*sICH criteria: ECASS II: ≥4pt ↑ NIHSS and any ICH; NINDS: any neurologic worsening due to any ICH; PROACT II: ≥4pt ↑ NIHSS at 36 h and any ICH; SITS-MOST: ≥4pt ↑ NIHSS at 24 h and PH2 HT*.

Further analysis of DIAS, DIAS 2, and DEDAS was pursued given the disparate results of Phase-2 trials (DIAS and DEDAS), suggesting efficacy and the negative efficacy results of Phase-3 trial (DIAS 2). In comparing the patient populations of the three trials, it was noted that there was a substantial difference between DIAS 2 and DIAS/DEDAS in the number of patients with intracranial vascular occlusion or high-grade stenosis (DIAS 2 30% vs. DIAS/DEDAS 57%; *P* ≤ 0.0001) (81). Moreover, in the pooled analysis of DIAS, DIAS 2, and DEDAS, desmoteplase treatment showed a favorable effect at 90 days in patients with either an intracranial vascular occlusion or high-grade stenosis (OR 4.14; 95% CI 1.40–12.23; *P* = 0.01) (81). Of note, favorable clinical response was defined as the composite of ≥8 point improvement in NIHSS (or 0–1), mRS < 3, and a Barthel Index Score ≥75 at 90 days. The subsequent randomized control trials DIAS-3 (82) and DIAS-4 (83) notably did not require infarct core–perfusion mismatch for enrollment, but only occlusion or stenosis of proximal segments of the middle, posterior, or anterior cerebral arteries and acute infarct lesion (on DWI or non-contrast CT) involving less than 1/3 MCA territory or 1/2 the anterior cerebral artery (ACA) or PCA territory. Both studies showed no safety concerns, but also no benefit 90-day functional outcomes (mRS < 3). Taken together, the results of the DIAS and DEDAS trials are mixed with regard to utilizing neuroimaging to select late-window stroke patients for treatment with thrombolytic therapy. On one hand, desmoteplase 3–9 h from LKW did not improve functional outcomes. However, a positive aspect of these studies was their demonstration that infarct core–perfusion mismatch can be effectively used in the emergent setting to efficiently triage acute stroke patients for potential treatment with thrombolytic therapy.

Around the same time that the DIAS 1–2 and DEDAS trials were underway to investigate desmoteplase with neuroimaging-based patient selection, several studies were simultaneously studying selection approaches for IV tPA using perfusion–diffusion mismatch. The Echoplanar Imaging Thrombolytic Evaluation Trial (EPITHET) was a Phase 2, observational trial of IV tPA in AIS patients 3–6 h from symptom onset (84). Out of 101 patients, 86% had perfusion–diffusion mismatch, using the same definition as DEFUSE. Of those patients that received IV tPA, there was decreased infarct growth (growth > 0%: 54% IV tPA vs. 77% placebo, *P* = 0.032) and increased reperfusion > 90% (56% IV tPA vs. 26% placebo, *P* = 0.01). Overall, however, there was no difference in 90-day mRS between the IV tPA and placebo groups (mRS < 3: 45% IV tPA vs. 40% placebo, *P* = 0.66). *Post hoc* analysis suggested that the previous failure of EPITHET was potentially due to too low a threshold for defining the PWI lesion (*T*_max_ > 2 s) (85) and subsequent studies by these investigators have used a stricter threshold of *T*_max_ > 6 s to define salvageable tissue.

An RCT used an alternative tissue plasminogen activator, tenecteplase, in AIS patients with infarct core–perfusion mismatch. This Phase-2B trial of tenecteplase for AIS, two doses of tenecteplase (0.1 or 0.25 mg/kg) administered within 6 h of stroke onset, was compared with IV tPA (86). Eligibility criteria included a CTP mismatch of greater than 20% and verified occlusion of an anterior, middle, or PCA. Twenty-five patients were randomized to each group. For the co-primary endpoints, there appeared to be a dose-dependent effect of tenecteplase on the proportion of the perfusion lesion reperfused (as assessed by PWI) and improvement in NIHSS at 24 h. In the pooled analysis, the tenecteplase group had higher rates of reperfusion at 24 h (79.3 vs. 55.4%; *P* = 0.004), improvement in 24-h NIHSS score (8.0 vs. 3.0; *P* < 0.001), reduced infarct growth at 90 days (2 vs. 12 cm^3^; *P* = 0.01), and increased rates of good functional outcome at 90 days (mRS < 2: 36 vs. 11%; *P* = 0.02) (86). These promising findings prompted Phase 3, randomized tenecteplase trial (NOR-TEST) of 1,100 adults with AIS in 13 centers in Norway (87). No difference between the 0.4-mg/kg tenecteplase and IV tPA groups was observed for the primary outcome of 90-day mRS of 0–1 (64 vs. 63%; *P* = 0.52). Importantly, in contrast to the prior Phase-2B trial, there were no imaging inclusion criteria of documented occlusion of an intracranial artery or any perfusion mismatch. As a result, 17% of enrolled patients were later confirmed as stroke mimics. Lastly, the randomized control trial of 0.25-mg/kg tenecteplase in patients with WUS, Tenecteplase in Wake-up Ischemic Stroke Trial (TWIST), is currently ongoing (88).

Similar studies have also been conducted using infarct core–perfusion mismatch criteria for patient selection for EVT. The Mechanical Retrieval and Recanalization of Stroke Clots Using Embolectomy (MR RESCUE) trial was the first trial initiated using the concept of core–perfusion mismatch for AIS patient triage (89). MR RESCUE was a Phase 2b, multicenter, randomized, open-label study of anterior circulation LVO patients, within 8 h of LKW, to EVT vs. usual medical care. Patients were stratified according to a favorable vs. non-favorable penumbral pattern that was defined as a predicted infarct core of <90 cm^3^ and proportion of predicted infarct tissue within region of interest as <70%. Unlike the definition of core–perfusion mismatch utilized in other trials, MR RESCUE employed a complex voxel-by-voxel algorithm requiring 4–7 variables on CTP or PWI (90). No difference was observed in mean 90-day mRS (3.9 vs. 3.9, *P* = 0.99); however, there were several important limitations important in considering the overall results of this trial. First, the trial used first-generation thrombectomy devices. Second, the trial had an exceedingly difficult time with enrollment, taking 7 years to enroll 118 patients across 22 high-volume stroke centers, likely due to a bias to randomize patients at enrolling sites. Next, in contrast to the subsequent positive EVT trials, subjects in MR RESCUE in the embolectomy, favorable penumbral arm had large estimated core volumes (median 36.2 cm^3^) and low rates (24%) of successful revascularization defined as a thrombolysis In Cerebral Infarction scale 2b/3. Lastly, the automated imaging program for penumbral stratification failed in 42% of cases.

**Figure 5:**
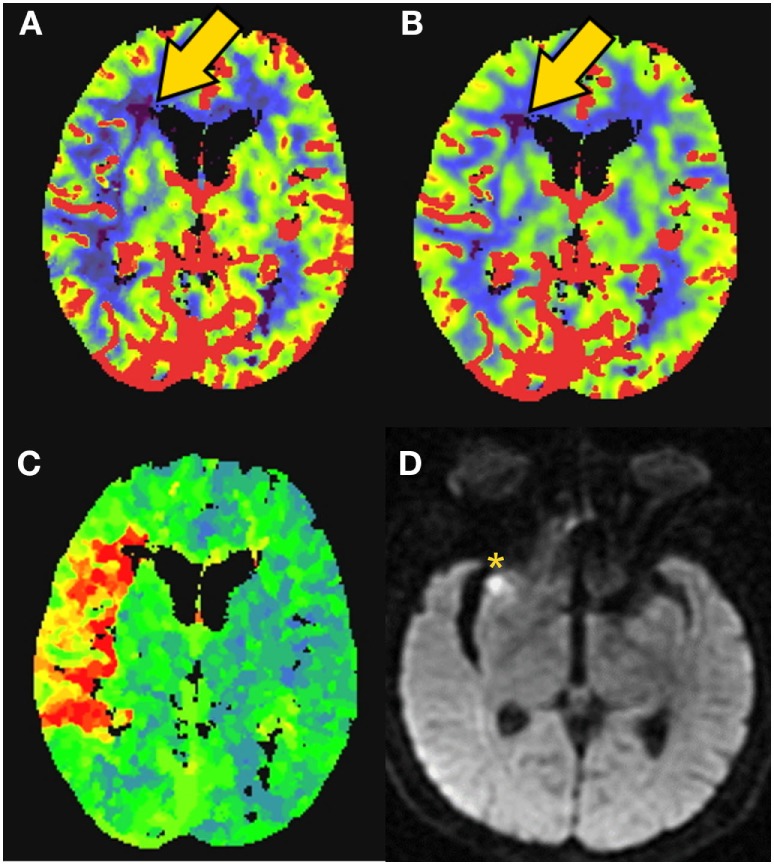
CT perfusion to identify salvageable tissue. 81-year-old female with right middle cerebral artery (MCA) syndrome and occlusion of the MCA on CT-angiogram. CT-perfusion maps: **(A)** cerebral blood flow (CBF), **(B)** cerebral blood volume (CBV), and **(C)** time to peak (TTP). Elevated time-to-peak contrast enhancement (TTP) colors orange to red represent >6-s delay **(C)**. Severely low blood flow and volume territories are violet color (gold arrow). The patient was rapidly revascularized and the final infarction is demonstrated on MRI diffusion weighted imaging [**(D)**––yellow asterisk]. Data analysis for figure was created under approval of local ethics committee.

EXTEND-IA used automated imaging analysis of CTP to select patients with occlusion of the intracranial ICA or first or second segment of the MCA and with salvageable tissue profile for EVT within 6 h from LKW. The mismatch profile was defined as follows: perfusion lesion *T*_max_ > 6 s, “infarct core” low CTP rCBF <30% normal tissue, low rCBF volume <70 cm^3^, mismatch ratio > 1.2, and absolute mismatch volume > 10 cm^3^. EVT initiated within 6 h of stroke onset and combined with mismatch for patient selection (see, e.g., Figure 5) significantly increased the likelihood of a favorable outcome (90-day mRS: generalized odds ratio, 2.0; 95%CI 1.2–3.8; 90-day mRS <3: 71 vs. 40%; *P* = 0.01) (5). EXTEND-IA demonstrated that early EVT, in combination with perfusion mismatch, was feasible for acute decision-making of LVO patients with SKSO. Moreover, in this trial EVT within 6 h of stroke onset was efficacious for reducing long-term disability.

These trials of late-window intravenous thrombolytic therapy and endovascular treatment have confirmed that advanced neuroimaging techniques can be employed in the emergent setting to triage acute stroke patients for acute therapies. As we will discuss below, these studies have prompted the application of infarct core–perfusion mismatch to the selection process of late-window or SUSO patients for revascularization therapies.

### Clinical–Core Mismatch

An alternative approach to treating patients with sufficient salvageable “penumbra” tissue to make the likely benefit of reperfusion therapy outweigh its risk is to treat patients with large clinical––“core” mismatches. In one study (91), the authors demonstrated that in 166 patients imaged within 12 h of onset with small “core” (DWI lesion ≤ 25 mL), but large clinical deficits (NIHSS ≥8) were more likely to experience early neurological deterioration (increase of NIHSS ≥4 points). Similar findings were found in 87 patients imaged within 24 h of LKW using DWI-ASPECTS ≥8 score to define core (92). Another study showed that such clinical–diffusion mismatches are also associated with perfusion–diffusion mismatch, with 93% specificity and 53% sensitivity in 54 patients imaged within 24 h of LKW (93). However, a separate study showed clinical–diffusion mismatch predicted perfusion–diffusion mismatch with only 65% sensitivity and 42% specificity in 68 patients (94). While another cohort study of 99 patients showed that clinical–diffusion mismatch was only 46% sensitive but 86% specific for perfusion–diffusion mismatch, benefits of IV tPA and reperfusion were similar in both patient groups with or without clinical–diffusion mismatch (95). On the other hand, in 43 EVT-eligible patients (M1 segment of the MCA-occlusion) with DWI lesions <25 mL, clinical–diffusion mismatch was found to be a better predictor of infarct growth than perfusion–diffusion mismatch (96). There have also been studies of non-contrast CT-based approaches for clinical–core mismatches. One investigation found no combination of CT ASPECTS and NIHSS predicted perfusion–diffusion mismatch (97) and another study found no relationship between “clinical–CT mismatch” and likelihood of responding to IV tPA (98). Researchers have also shown that EVT decisions were changed rarely (5.6%) when including CTP in addition to NIHSS, non-contrast CT and CTA (99).

SWIFT PRIME, a prospective, randomized open-label clinical trial, which showed benefit for EVT for anterior circulation LVO stroke patients within 6 h of LKW also employed a modified clinical––“core” mismatch for part of the study (4). Patients were originally excluded based on MRI- or CT-assessed infarct core >50 cm^3^, ischemic penumbra <15 cm^3^, and mismatch ratio <1.8. After enrollment of the first 71 patients in SWIFT PRIME using infarct core–perfusion mismatch as part of its inclusion criteria, the approach was modified to accommodate study sites without perfusion imaging capabilities based only on the extent of ischemic changes on CT (ASPECTS ≥6) (4). Thirty-seven patients were enrolled with this modified inclusion criteria based on core size. Patients treated with Stent Retriever plus IV tPA were significantly more likely to be functionally independent at 90 days (mRS < 3: 60 vs. 35%; risk ratio 1.70, 95%CI 1.23–2.33; *P* < 0.001).

The results of these studies provide evidence that clinical––“core” mismatch can function as an indicator of potentially salvageable tissue in the decision-making processes of acute stroke. A major advantage of this approach for patient triage is the independence from relying on perfusion imaging, which is not universally available at all hospitals that treat AIS patients. The best evidence for benefit in the late-window (beyond 6 h for EVT) currently relies on core volume estimates either by DWI, or by CT-rCBF, which implies that stroke centers of all levels will eventually need to become facile with some form of advanced imaging in late-window patients. In later sections, we will discuss the recent clinical trials that used this approach to expand the window of eligibility for thrombolysis in SUSO patients.

### Collateral Grade

A third approach for selecting patients for late thrombolysis relies on the status of the pial collaterals. This is also a pragmatic approach for EVT candidates since all patients are screened with vessel imaging to identify LVO that obviates additional imaging. Studies have shown that patients with a malignant CTA profile, defined as the absence of collaterals in >50% of an MCA M2 branch, also have large DWI lesions (100). A retrospective analysis of the IMS III trial of 95 patients with both diagnostic-quality CTA and CTP showed that patients presenting with good collaterals tend to have smaller cores and greater perfusion mismatch (101). In addition, in 276 patients with CTA, robust collaterals were associated with good clinical outcomes (102). However, another study of 60 patients imaged within 12 h of LKW showed that patients with target perfusion–diffusion mismatch did well irrespective of collateral score (103). In ESCAPE, which was the Canadian multicenter randomized Phase-3 trial of EVT for LVO in 316 patients up to 12 h from LKW, a notable, distinct inclusion criterion was evidence of moderate-to-good collateral circulation of the MCA territory on multiphase CTA (48). Importantly, 6.3% of participants enrolled had evidence of poor collateral circulation on analysis by the core laboratory. Although patients could be enrolled up to 12 h from LKW, the median time from LKW to reperfusion was approximately 4 h, with only 49 subjects randomized after 6 h, and thus the ESCAPE study cannot be considered a comprehensive study of late revascularization intervention.

These observations suggest that collateral status can function as another imaging surrogate of salvageable tissue. Specifically, patients with good pial collaterals are more likely capable of sustaining salvageable tissue for relatively longer periods of time and thereby could be candidates for extended window therapeutic interventions. Collateral status is likely an important variable in determining the rate of tissue death over time in hypoperfused brain. While there is a correlation between collateral status and CTP (101, 104), it is unclear whether collateral status is superior to CTP in the triage of SUSO patients.

## Retrospective Studies of Off-Label Revascularization Treatment of SUSO Patients

Because of encouraging studies characterizing SUSO patients and suggesting a potential benefit of reperfusion therapy due to similarity in imaging presentation with early witnessed stroke patients; there have been several retrospective analyses of patients who were treated with off-label IV tPA or EVT (Table 2) based on imaging techniques described previously. We will discuss briefly the safety and efficacy findings in these retrospective studies that paved the way for the pivotal prospective trials of extended window intervention of AIS patients.

**Table 2:** Retrospective studies of off-label revascularization treatment of SUSO patients.

Study	N	SxD (h)	Arms	Imaging selection	Outcome
Cho AH (105)	32	3–6	IA, IV SUSO vs. SKSO	MRI (DWI/PWI/FLAIR mismatch)	Rates of recanalization, early neurological improvement and 90-day outcome comparable.

Barreto A (106)	46	ND	IA, IV, IV + IA WUS vs. non-lysed WUS	CT (<1/3 MCA)	Treated WUS better outcome than non-treated WUS but higher mortality.

Manawadu D (107)	68	4.5	WUS vs. on-label IV tPA	CT (<1/3 MCA)	90-day favorable outcome (mRS ≤ 2), sICH rates not significantly different

Jovin TG (110)	237	8–24	EVT	MRI (DWI/FLAIR/PWI mismatch) or CTP	Acceptable safety for EVT beyond 8 h of stroke onset.

Aghaebrahim A (109)	78	4.5	WUS vs. witnessed stroke > 8-h EVT	CT or MRI (ASPECTS > 6, <1/3 MCA)	90-day favorable outcome (mRS ≤ 2), PH and final infarct volumes not significantly different

*CT, computed tomography; DWI, diffusion-weighted imaging; EVT, endovascular thrombectomy; FLAIR, fluid attenuated inversion recovery; IA, intra-arterial; ICH, intracranial hemorrhage; IV, intravenous; MCA, middle cerebral artery; MRI, magnetic resonance imaging; mRS, modified Rankin scale; PH, parenchymal hematoma; PWI, perfusion weighted imaging; sICH, symptomatic intracranial hemorrhage; SKSO, stroke of known symptom onset; SUSO, stroke of unknown symptom onset; WUS, wake-up stroke*.

### Intravenous Thrombolysis

A retrospective analysis of 32 SUSO patients treated with thrombolytic therapy at 3 Korean medical centers using MRI specific eligibility criteria (perfusion–diffusion mismatch > 20% MTT to DWI, no FLAIR changes, and DWI volume <50% MCA territory) showed that an MRI-based algorithm for thrombolysis of SUSO patients was feasible. In comparing the SUSO to SKSO groups, baseline characteristics were similar, including age and admission NIHSS scale, and no difference was observed in rates of recanalization (immediate 81.3 vs. 63.1%; *P* = 0.06; delayed 80.6 vs. 69.1%; *P* = 0.28), 90-day mRS ≤ 2 (50 vs. 49.3%, *P* = 1.0), or sICH (6.3 vs. 5.8%; *P* = 1.0) (105).

In another retrospective single-center study of thrombolytic therapy for WUS, administered on a compassionate basis, criteria for thrombolytic therapy in the WUS cohort included the following: (1) patients were neurologically normal before going to sleep, (2) patients awakened with a disabling deficit, and (3) CT head showed no hypodensity exceeding 1/3 the MCA (106). Forty-six WUS patients that received thrombolytic therapy were identified, of which 61% were treated with IV tPA and 30% with only EVT and the remaining receiving combination treatment (106). In the thrombolysed WUS group, two patients experienced sICH (4.3% thrombolysed WUS vs. 0% non-treated WUS). Despite higher mortality (15 vs. 0%) compared with non-treated WUS patients, thrombolysed WUS patients were more likely to experience a favorable outcome (90-day mRS 0–2: 28 vs. 13%, *P* = 0.006) (106). Compared with 174 standard-of-care 0 to 3-h IV tPA-treated patients, treated WUS patients had higher rates of sICH (4.3 vs. 2.9%; *P* = 0.64) and a lower, but statistically insignificant, likelihood of favorable outcome (28 vs. 48%, *P* = 0.64).

A retrospective analysis of 68 WUS patients presenting within 4.5–12 h from LKW and treated with thrombolytic therapy from another center showed no difference in the number of patients achieving a 90-day mRS of 0–2 (38 vs. 37%, *P* = 0.89) or rate of symptomatic ICH (sICH: 3.4 vs. 2.9%, *P* = 1.0) compared with 326 patients receiving IV tPA within 4.5 h of symptom onset (107). Notable inclusion criteria for the WUS group included: NIHSS ≥5 and no or early ischemic changes <1/3 MCA territory as assessed by ASPECTS (107).

### Endovascular Treatment

With regard to EVT, a retrospective, single-center study of EVT without any advanced neuroimaging in WUS patients has also been reported. In 213 LVO anterior circulation ischemic stroke patients that underwent EVT after being deemed ineligible for IV tPA, including 21 WUS patients and 33 patients treated beyond 8 h from LKW, an increased odds of sICH (14.3%; odds ratio = 4.9, 95%CI 1.03–23.6; *P* = 0.047) in WUS patients compared with the group treated within 8 h of symptom onset (6.7%; odds ratio 3.8, 95%CI 1.07–13.7; *P* = 0.04) (108). Despite this observation, the authors reported no difference in the 90-day mRS between the WUS and group treated within 8 h of stroke onset (108). Another retrospective, single-center review of EVT comparing outcomes between 78 WUS patients and 128 late-window (beyond 8 h from LKW) SKSO patients who presented with small core and large perfusion defect found similar results (109). No significant difference was observed in baseline NIHSS, rates of successful recanalization, 90-day mRS ≤ 2 (43 vs. 50%, *P* = 0.3), parenchymal hematoma (9 vs. 5.5%; *P* = 0.3), or final infarct volume (75.2 vs. 61.4 cm^3^; *P* = 0.6).

A multicenter, retrospective analysis of patients with LVO (EVT initiated beyond 8 h from LKW) and perfusion imaging used for selection criteria suggested feasibility and potential efficacy of late-window EVT (110). In 237 patients meeting those inclusion criteria the mean treatment time was 15 h from LKW. Forty-five percent of the patients achieved a good functional outcome at 90-days or time of hospital discharge (mRS < 3). Parenchymal hematoma occurred in 8.9% of the patients and the 90-day mortality rate was 21.5%.

In addition to perfusion imaging, the status of the pial collateral circulation has also been evaluated as a potential metric for extending the window for EVT eligibility. A retrospective, single-center study of 61 anterior circulation LVO patients showed that in contrast to patients with poor collateral status, patients with good pial collaterals had no temporal cutoff point for total time of ischemia and predicting clinical improvement (111). In comparing good vs. poor collateral status, clinical improvement (4-point decline in NIHSS from baseline to discharge) beyond 300 min was significantly higher in the group with good pial collaterals (90.1 vs. 23.1%; *P* = 0.010). In agreement with these findings, another retrospective analysis of 237 patients with anterior circulation LVOs undergoing EVT also demonstrated that in patients with good collateral grades the probability of favorable outcome is not significantly influenced by onset-to-reperfusion time (112).

The interpretation of these retrospective studies is limited by the retrospective nature and inconsistency in neuroimaging selection criteria. Nonetheless, these findings demonstrate that neuroimaging-based triage for EVT is feasible and safe beyond 8 h from LKW and prompted the development of several prospective studies to further assess for efficacy.

## Prospective Clinical Trials of Revascularization Therapies for SUSO Patients

### Intravenous Thrombolysis

Based on the promising findings of retrospective studies of revascularization interventions for SUSO patients, several prospective studies have been launched (Table 3). In 2003, one of the earliest studies involved abciximab, which had a prespecified cohort of WUS patients, although the primary cohort involved patients that could be treated within 5 h of stroke onset. Phase-3 RCT of abciximab (AbESTT-II), which is a platelet glycoprotein IIb/IIIa inhibitor, was terminated early in 2005 due to a significantly increased rate of symptomatic and fatal ICH (5.5% of abciximab-treated vs. 0.5% placebo, *P* = 0.002) (113). Of the WUS cohort (43 patients, 22 treated with abciximab, 21 treated with placebo), there was no improvement in 90-day mRS and an increased rate of symptomatic and fatal ICH at 5 days (13.6 vs. 5% placebo, *P* = 0.347) and 3 months (18.2 vs. 5%, *P* = 0.193) Secondary analysis showed that the WUS cohort who received abciximab tended to have greater rates of new strokes on baseline CT and bleeding but otherwise were comparable to other patients in the study (114).

**Table 3:** Prospective trials of thrombolysis in WUS and non-WUS SUSO patients.

Study	Phase	*N*	SxD (h)	Design	Study drug	Imaging selection	sICH definition	sICH (%)	Primary outcome
AbESTT-II^a^	3	808	3	Two arms	Abciximab, placebo	CT (<50% MCA)	NINDS	5.5	90-day mRS adjusted for stroke severity: 32% vs. 33%.

Wake-up Stroke^e^	2	40	3	Open label	IV tPA	CT (<1/3 MCA)	ECASS III	0	sICH; 52.6% 90-day mRS < 2

Aoki (118)	NA	10	3	Open label, Single arm	IV tPA	MRI (DWI/FLAIR signal intensity ratio)	ECASS III	0	90-day favorable outcome (mRS ≤ 2) found in four patients.

SAIL-ON^e^	2	20	4.5	Open label	IV tPA	CT or MRI (<1/3 MCA)	ECASS II NINDS	0	sICH

RESTORE^d^	2	83	6	Open label, Single arm	IV tPA/IV + IA UK or IA UK	MRI (DWI/PWI/FLAIR)	ECASS II, NINDS	3.6	90-day mRS 0–2: 44.6%.

MR WITNESS	2	80	4.5	Open label	IV tPA	MRI (DWI/FLAIR signal intensity ratio)	ECASS III	1.25	sICH

WAKE-UP^c,e^	3	800	4.5	Two arms	IV, placebo	MRI (DWI/FLAIR mismatch)	ECASS II, SITS-MOST, NINDS	NA	90-day mRS 0–1

THAWS	3	300	4.5	Two arms	IV, placebo	MRI (DWI/FLAIR mismatch)	ECASS II, SITS-MOST, NINDS	NA	90-day mRS 0–1 in Japanese stroke patients.

DAWN^b^	2/3	206	6–24 h	Two arms	EVT	CT or MRI (<1/3 MCA, ICA/M1 occlusion, clinical/NIHSS mismatch)	ECASS III	6	90-day mRS 0–2: 48.6% vs. 13.1%.

DEFUSE 3^b^	3	182	6–16 h	Two arms	EVT	ICA/M1 occlusion, target mismatch	ECASS II	7	90-day mRS 0–2: 45% vs. 17%.

*^a^Terminated (808 enrolled)*.

*^b^Halted for overwhelming efficacy*.

*^c^Halted for funding stoppage*.

*^d^Treatment group compared with registry of controls*.

*^e^Trial enrolled on wake-up stroke patients*.

*CT, computed tomography; DWI, diffusion-weighted imaging; EVT, endovascular thrombectomy; FLAIR, fluid attenuated inversion recovery; IA, intra-arterial; ICH, intracranial hemorrhage; IV, intravenous; MCA, middle cerebral artery; MRI, magnetic resonance imaging; mRS, modified Rankin scale; PH, parenchymal hematoma; PWI, perfusion weighted imaging; sICH, symptomatic intracranial hemorrhage*.

In 2013, another WUS investigation, Wake-up Stroke, completed involving a single-arm prospective open-label, multicenter safety trial of 40 WUS patients treated with IV tPA within 3 h of symptom discovery (115). The median NIHSS of this cohort was 6.5 and IV tPA was administered at a mean time of 10.3 ± 2.6 h from LKW. No sICH occurred in this population and 52.6% had an excellent functional outcome at 90 days (mRS 0–1) (115). While this trial is limited by its relatively small sample size, lack of control group, and open-label design, the strength of this trial is its pragmatic triage requirement of only a non-contrast head CT. A similarly designed prospective open-label, multicenter safety trial of IV tPA treatment within 4.5 h of symptom discovery of 20 WUS patients, Safety of intravenous thrombolytics in stroke on awakening (SAIL-ON), also reported no sICH (116). Both these trials enrolled stroke mimics, which might have contributed to the high rates of good outcome.

In parallel with the CT trials, MRI-based trials were underway investigating imaging-selected revascularization of SUSO patients. Launched in 2006, RESTORE (Reperfusion therapy in unclear-onset stroke based on MRI evaluation) was a prospective, multicenter, single-arm trial of SUSO patients with thrombolytic therapy within 6 h of symptom discovery (117). Patients were included if presenting with perfusion–diffusion mismatch, but excluded if FLAIR hyperintensities were noted. Out of 430 SUSO patients, 83 patients were treated with thrombolytic therapy including 63 WUS patients (117). Of those treated SUSO patients, the median LKW to hospital presentation was 8.6 h (interquartile range 5.4–11.1 h). In total, 89.2% of patients had an LVO and 68.7% of patients received only intra-arterial therapy, which included intra-arterial urokinase mechanical clot disruption, or angioplasty. At 3 months, 44.6% of patients had an mRS < 2 and only 3.6% had sICH. Compared with the non-treated registry-based control group, the treated group had increased odds of good outcome (mRS 0–2: OR 2.25; 95% CI 1.14–4.49) suggesting a potential benefit of revascularization therapy in this population. However, the interpretation and generalizability of these results are limited due to the use of registry patients as the control group. The RESTORE trial can be considered more aptly an investigation of perfusion–diffusion mismatch enrollment criteria, with an additional restriction of FLAIR negativity.

There have been trials that investigated directly the concept of using DFM for patient selection. In 2009, there was a small prospective trial of IV tPA (0.6 mg/kg) of SUSO patients with ICA or MCA M1 or M2 occlusions based on DFM who could be treated within 3 h of symptom discovery [fluid attenuated inversion recovery imaging-based intravenous recombinant tissue plasminogen activator (rt-PA) therapy study] (118). Ten subjects were enrolled, of which four were WUS. Favorable outcome was defined as mRS 0–2. No sICH was observed in this group and seven of the patients had recanalization at 7 days after IV tPA administration. Favorable outcome was observed in 40% of subjects. Notably 30% of the subjects had prestroke mRS greater than 2. In 2011, the MR WITNESS trial (A Study of Intravenous Thrombolysis with Alteplase in MRI-Selected Patients), a Phase 2a, open-label multicenter trial of IV tPA (0.9 mg/kg) in SUSO patients with DFM 4.5–24 h from LKW, launched (119). This trial enrolled 80 subjects with a primary safety outcome of sICH in only 1 subject, and a rate of excellent functional outcome at 90 days (mRS 0–1) of 44% among the 69 subjects with a prestroke mRS of 0–1. In summary, these findings suggest that the administration of IV tPA to SUSO patients within 3 h of symptom discovery is safe and feasible.

Two large, prospective clinical trials assessing DFM in the triage of AIS patients are in progress or recently completed that address efficacy issues. The Thrombolysis for Acute Wake-up and unclear-onset Strokes (THAWS) is a multicenter, prospective, open-label trial currently enrolling in Japan that is investigating a lower dose of IV tPA (0.6 mg/kg, which is the approved dose for Japanese stroke patients) in patients with stroke onset 4.5–12 h from LKW and DFM on MRI (120). The anticipated enrollment is 300 patients and the primary outcome measures are 90-day mRS < 2 and sICH. WAKE-UP (Efficacy and Safety of MRI-Based Thrombolysis in Wake-Up Stroke: A Randomized, Double-blind, Placebo-controlled Trial) is a European multicenter, randomized placebo-controlled, Phase-3 trial using DFM as criterion for IV tPA treatment of AIS patients with > 4.5 h LKW (30). This study was stopped due to lack of funding and results are anticipated this year. Out of 1,362 patients enrolled, 503 were randomized (planned 800) and 859 participants were screen failures. In Tables 2 and 3, we summarize the major prospective and retrospective studies on thrombolytic therapy of SUSO including WUS.

In addition to DFM trials, there is also a large Phase-3 trial Extending the time for Thrombolysis in Emergency Neurological Deficits (EXTEND) trial which uses infarct core–perfusion mismatch in patients 3 or 4.5–9 h from LKW or WUS within 9 h from midpoint of sleep duration to determine eligibility for treatment with IV tPA (121). The neuroimaging inclusion criteria of this trial are (1) a small “infarct core” defined as DWI or CT-rCBF lesion volume <70 cm^3^ and (2) core–perfusion mismatch > 1.2 and absolute mismatch > 10 cm^3^. The perfusion lesion is defined on PWI or CTP as *T*_max_ > 6 s. The primary outcome is rate of 90-day mRS 0–1 outcomes in the IV tPA group compared with the placebo group. The expected enrollment is 400 patients with an anticipated completion date of 2019.

While the current evidence for pretreatment advanced neuroimaging to guide decision-making for IV thrombolytic therapies in SUSO patients is intriguing, at present, there is no positive Phase-3 trial to warrant the routine use in clinical practice. This statement is supported by the 2018 American Heart Association/American Stroke Association Guidelines recommendation of no benefit to this approach (11). There is therefore a clinical opportunity to expand IV tPA to more patients outside the current approved treatment window of 4.5 h from LKW with such a trial.

### Endovascular Treatment

The recently published DAWN trial represents the first randomized, multicenter, Phase-3 trial utilizing an automated neuroimaging approach to triage late-window LVO patients for EVT (8). Distinct from the previously discussed studies, DAWN employed the concept of clinical–ischemic core mismatch to identify LVO patients with occlusion of the intracranial ICA and/or first segment of the MCA that were hypothesized to benefit from EVT. The inclusion criteria were therefore a combination of NIHSS and age-dependent infarct volume assessed by DWI or rCBF volume. Specifically, LVO patients 6–24 h from LKW and age less than 80 years were eligible if infarct volume was <31 cm^3^ and NIHSS ≥10 or infarct volume was 31–51 cm^3^ and NIHSS ≥20. For LVO patients greater than 80 years of age, inclusion criteria were NIHSS ≥10 and infarct volume <21 cm^3^ (8). A total of 206 patients were enrolled in the trial with 107 randomized to EVT. The median time from LKW to randomization was 12.2 h in the EVT group. The trial was stopped early for overwhelming efficacy according to a prespecified interval assessment. At 90 days, 49% of the EVT group vs. 13% of the standard medical therapy group achieved functional independence (mRS < 3; 95% credible interval 24–44; posterior probability of superiority, > 99.9%). There was no difference in the rate of sICH (6 vs. 3%; *P* = 0.5) or 90-day mortality (19 vs. 18%; *P* = 1.0) in the EVT group compared with standard medical therapy (8).

The DEFUSE-3 trial also showed substantial benefit of EVT from an infarct core–perfusion mismatch strategy of LVO patient selection 6–16 h after onset (7). DEFUSE 3 used the same neuroimaging definition of core–perfusion mismatch as DEFUSE 2 using MRI or CT-rCBF (infarct core < 70 cm^3^, mismatch ratio ≥1.8, mismatch volume ≥15 cm^3^) in patients with anterior circulation LVO (defined as ICA or M1 segment MCA). Importantly, as a result of DAWN, the trial was halted prematurely for an interim analysis, which exceeded the efficacy endpoint. Of the 92 patients that were randomized to EVT, 53% were WUS and 75% received CTP to assess for core–perfusion mismatch. Both the EVT and standard medical therapy groups had small ischemic cores (9.4 cm^3^ EVT vs. 10.1-cm^3^ medical therapy) and large hypoperfused areas (114.7-cm^3^ EVT vs. 116.1-cm^3^ medical therapy). EVT plus standard medical therapy significantly reduced disability assessed by 90-day mRS (unadjusted common odds ratio 2.77, 95%CI 1.63–4.70; mRS 0–2 45% EVT vs. 17% standard medical therapy, *P* < 0.001). There was no difference in sICH between groups (7 vs. 4%; *P* = 0.75).

There are several additional points of DEFUSE 3 that further inform on the approach of core–perfusion mismatch in the triage of late-window LVO patients. First, of the 296 patients originally consented, 107 patients did not fulfill imaging inclusion criteria (36.1%). Secondly, 70 patients included in DEFUSE 3 would have been ineligible for DAWN, largely based on ischemic core size; however, the DAWN-ineligible group showed a similar benefit for late-window EVT (odds ratio 2.96, 95%CI 1.26–6.97). This observation speaks to the potential for core–perfusion mismatch to expand eligibility for reperfusion therapies beyond clinical–core mismatch. Third, WUS patients also showed a similar benefit of EVT (odds ratio 3.44, 95% CI 1.60–7.38). Lastly, and somewhat surprisingly, at 24-h post-revascularization therapy there was no significant difference in median infarct volume between groups (35-cm^3^ EVT vs. 41-cm^3^ medical therapy; *P* = 0.19). The underlying explanation for this observation is unclear and certainly warrants further investigation given the dramatic benefit of EVT on functional outcomes.

The results of DAWN and DEFUSE 3 are highly impactful since they will significantly alter the management and triage of patients previously thought to be “out of the window” for reperfusion therapy. In fact, the updated 2018 American Heart Association/American Stroke Association guidelines for management of anterior circulation LVO patients 6–24 h from LKW now recommend (level IA) obtaining CTP, DWI sequences or PWI to assist in patient selection for EVT (11). The findings of DAWN and DEFUSE 3 confirm that a subpopulation of LVO patients beyond 6 h from LKW with salvageable tissue, as evidenced by a clinical–ischemic core mismatch, that still benefit from EVT. In addition, while DAWN used CTP to determine the infarct core in a subset of patients, there was no core–perfusion mismatch requirement. There are, however, several additional findings from DAWN that merit discussion with regard to the overall generalizability of these findings. First, it is unknown how many patients were screened to enroll the 206 subjects reported in DAWN as screening logs were not collected. The discrepancy between the median NIHSS of 17 (interquartile range 13–21) in the EVT group but a median infarct volume of only 7.6 cm^3^ despite an LVO suggests a prolonged phase of penumbral survival and an opportunity to intervene may be present in more subjects than previously thought. These findings reinforce the critical role of collaterals in sustaining salvageable tissue until thrombolytic therapy is possible and affirm the inclusion criteria of ESCAPE. The poor outcomes seen in the medical arm of DEFUSE 3 and DAWN suggest that delayed collateral failure is common. The second important issue is how to generalize the results of DAWN and DEFUSE 3 to routine clinical practice. Future research should examine what additional subgroups of late-window LVO subjects can benefit from EVT. TENSION (Efficacy and safety of ThrombEctomy IN Stroke with extended lesion and extended time window) is one example of such a trial that plans to evaluate whether patients with severe strokes and large core volume (estimated by ASPECTS) can still have a relative benefit from EVT (122). TENSION is a prospective, open label, blinded endpoint, European randomized trial comparing the effectiveness of EVT in LVO patients with large infarcts (ASPECTS 3–5) up to 12 h or unknown LKW using an mRS ordinal analysis.

## Conclusion

The benefits of reperfusion therapies for acute ischemic stroke are well established for appropriately selected patients based on the duration of stroke symptoms. Neuroimaging-based methods of patient selection have, however, demonstrated the ability to identify additional populations of stroke patients that could benefit from late-window reperfusion therapy. Advanced neuroimaging techniques are both feasible and efficacious in the treatment allocation of SUSO patients based on either the presence of salvageable tissue on clinical-imaging mismatch or *via* a radiographic time-stamp of stroke duration. Going forward, with the anticipated results of several large Phase 3 trials, the management of this unique population of stroke patients will likely change for the better. Future research should continue to refine the approach to identifying additional populations of SUSO patients that would benefit from reperfusion therapy.

## Author Contributions

ME conceived the idea, drafted the manuscript, designed the figures, and provided critical review of the manuscript and figures. AB designed the figures, and provided critical review of the manuscript and figures. LS provided critical review of the manuscript and figures. OW conceived the idea, drafted the manuscript, designed the figures, and provided critical review of the manuscript and figures.

## Conflict of Interest Statement

ME reports no disclosures. OW is the co-inventor of a patent on Delay-compensated calculation of tissue blood flow, US Patent 7,512,435. 31 March 2009, and the patent has been licensed to General Electric, Siemens, Imaging Biometrics and Olea Medical, consultant to Penumbra on topics unrelated to the article. OW and LS report being the principal investigator of an investigator-initiated study of extended-window intravenous thrombolysis funded by the National Institutes of Neurological Disorders and Stroke (clinicaltrials.gov/show/NCT01282242) for which Genentech provides alteplase free of charge to Massachusetts General Hospital as well as supplemental per-patient payments to participating sites. LS reports serving as chair of the AHA/ASA GWTG stroke clinical work group and hospital accreditation Science Committee; serving as a stroke systems consultant to the Massachusetts Department of Public Health; and serving as a scientific consultant to LifeImage regarding user interface design and usability, and regarding trial design and conduct to Lundbeck (international steering committee, DIAS3, 4 trial), Penumbra (data and safety monitoring committee, Separator 3D and MIND trials) and Medtronic (Victory AF and Stroke AF trials). AB reports that the Wake-up Stroke study was partially supported by Genentech Inc., which provided study medication without charge as well as an investigator initiated grant to another investigator for monetary support of enrollment and pharmacy fees.

## References

[1] Hacke W Kaste M Bluhmki E Brozman M Davalos A Guidetti D Thrombolysis with alteplase 3 to 4.5 hours after acute ischemic stroke. N Engl J Med (2008) 359(13):1317–29. 10.1056/NEJMoa0804656 18815396

[2] National Institute of Neurological Disorders and Stroke rt-PA Stroke Study Group. Tissue plasminogen activator for acute ischemic stroke. N Engl J Med (1995) 333(24):1581–7. 10.1056/NEJM199512143332401 7477192

[3] Goyal M Menon BK van Zwam WH Dippel DW Mitchell PJ Demchuk AM Endovascular thrombectomy after large-vessel ischaemic stroke: a meta-analysis of individual patient data from five randomised trials. Lancet (2016) 387(10029):1723–31. 10.1016/S0140-6736(16)00163-X 26898852

[4] Saver JL Goyal M Bonafe A Diener HC Levy EI Pereira VM Stent-retriever thrombectomy after intravenous t-PA vs. t-PA alone in stroke. N Engl J Med (2015) 372(24):2285–95. 10.1056/NEJMoa1415061 25882376

[5] Campbell BC Mitchell PJ Kleinig TJ Dewey HM Churilov L Yassi N Endovascular therapy for ischemic stroke with perfusion-imaging selection. N Engl J Med (2015) 372(11):1009–18. 10.1056/NEJMoa1414792 25671797

[6] Berkhemer OA Fransen PS Beumer D van den Berg LA Lingsma HF Yoo AJ A randomized trial of intraarterial treatment for acute ischemic stroke. N Engl J Med (2015) 372(1):11–20. 10.1056/NEJMoa1411587 25517348

[7] Albers GW Marks MP Kemp S Christensen S Tsai JP Ortega-Gutierrez S Thrombectomy for stroke at 6 to 16 hours with selection by perfusion imaging. N Engl J Med (2018) 378(8):708–18. 10.1056/NEJMoa1713973 29364767 PMC6590673

[8] Nogueira RG Jadhav AP Haussen DC Bonafe A Budzik RF Bhuva P Thrombectomy 6 to 24 hours after stroke with a mismatch between deficit and infarct. N Engl J Med (2018) 378(1):11–21. 10.1056/NEJMoa1706442. 29129157

[9] Jadhav AP Desai SM Kenmuir CL Rocha M Starr MT Molyneaux BJ Eligibility for endovascular trial enrollment in the 6- to 24-hour time window: analysis of a single comprehensive stroke center. Stroke (2018) 49(4):1015–7. 10.1161/STROKEAHA.117.020273 29581344

[10] Adeoye O Hornung R Khatri P Kleindorfer D. Recombinant tissue-type plasminogen activator use for ischemic stroke in the United States: a doubling of treatment rates over the course of 5 years. Stroke (2011) 42(7):1952–5. 10.1161/STROKEAHA.110.612358 21636813 PMC4114342

[11] Powers WJ Rabinstein AA Ackerson T Adeoye OM Bambakidis NC Becker K 2018 guidelines for the early management of patients with acute ischemic stroke: a guideline for healthcare professionals from the American Heart Association/American Stroke Association. Stroke (2018) 49(3):e46–110. 10.1161/STR.0000000000000158 29367334

[12] Maas MB Singhal AB. Unwitnessed stroke: impact of different onset times on eligibility into stroke trials. J Stroke Cerebrovasc Dis (2013) 22(3):241–3. 10.1016/j.jstrokecerebrovasdis.2011.08.004 21917477 PMC3718254

[13] Kim YJ Kim BJ Kwon SU Kim JS Kang DW. Unclear-onset stroke: daytime-unwitnessed stroke vs. wake-up stroke. Int J Stroke (2016) 11(2):212–20. 10.1177/1747493015616513 26783313

[14] Fink JN Kumar S Horkan C Linfante I Selim MH Caplan LR The stroke patient who woke up: clinical and radiological features, including diffusion and perfusion MRI. Stroke (2002) 33(4):988–93. 10.1161/01.STR.0000014585.17714.67 11935049

[15] Marler JR Price TR Clark GL Muller JE Robertson T Mohr JP Morning increase in onset of ischemic stroke. Stroke (1989) 20(4):473–6. 10.1161/01.STR.20.4.473 2648651

[16] Mackey J Kleindorfer D Sucharew H Moomaw CJ Kissela BM Alwell K Population-based study of wake-up strokes. Neurology (2011) 76(19):1662–7. 10.1212/WNL.0b013e318219fb30 21555734 PMC3100086

[17] Wu O Schwamm LH Sorensen AG. Imaging stroke patients with unclear onset times. Neuroimaging Clin N Am (2011) 21(2):327–44, xi. 10.1016/j.nic.2011.02.008 21640303 PMC3109317

[18] Chaturvedi S Adams HP Jr Woolson RF. Circadian variation in ischemic stroke subtypes. Stroke (1999) 30(9):1792–5. 10.1161/01.STR.30.9.1792 10471425

[19] Serena J Davalos A Segura T Mostacero E Castillo J. Stroke on awakening: looking for a more rational management. Cerebrovasc Dis (2003) 16(2):128–33. 10.1159/000070592 12792170

[20] Lago A Geffner D Tembl J Landete L Valero C Baquero M. Circadian variation in acute ischemic stroke: a hospital-based study. Stroke (1998) 29(9):1873–5. 10.1161/01.STR.29.9.1873 9731611

[21] Argentino C Toni D Rasura M Violi F Sacchetti ML Allegretta A Circadian variation in the frequency of ischemic stroke. Stroke (1990) 21(3):387–9. 10.1161/01.STR.21.3.387 2309262

[22] Elliott WJ. Circadian variation in the timing of stroke onset: a meta-analysis. Stroke (1998) 29(5):992–6. 10.1161/01.STR.29.5.992 9596248

[23] Andrews NP Gralnick HR Merryman P Vail M Quyyumi AA. Mechanisms underlying the morning increase in platelet aggregation: a flow cytometry study. J Am Coll Cardiol (1996) 28(7):1789–95. 10.1016/S0735-1097(96)00398-1 8962568

[24] Redon J The normal circadian pattern of blood pressure: implications for treatment. Int J Clin Pract Suppl (2004) 145:3–8. 10.1111/j.1742-1241.2004.00403.x 15617452

[25] Scheer FA Shea SA. Human circadian system causes a morning peak in prothrombotic plasminogen activator inhibitor-1 (PAI-1) independent of the sleep/wake cycle. Blood (2014) 123(4):590–3. 10.1182/blood-2013-07-517060 24200683 PMC3901072

[26] Todo K Moriwaki H Saito K Tanaka M Oe H Naritomi H. Early CT findings in unknown-onset and wake-up strokes. Cerebrovasc Dis (2006) 21(5–6):367–71. 10.1159/000091545 16490949

[27] Denny MC Boehme AK Dorsey AM George AJ Yeh AD Albright KC Wake-up strokes are similar to known-onset morning strokes in severity and outcome. J Neurol Neurol Disord (2014) 1(1):102. 10.15744/2454-4981.1.102 26835514 PMC4732736

[28] Moradiya Y Janjua N Presentation and outcomes of “wake-up strokes” in a large randomized stroke trial: analysis of data from the international stroke trial. J Stroke Cerebrovasc Dis (2013) 22(8):e286–92. 10.1016/j.jstrokecerebrovasdis.2012.07.016 22939198

[29] Reid JM Dai D Cheripelli B Christian C Reidy Y Gubitz GJ Differences in wake-up and unknown onset stroke examined in a stroke registry. Int J Stroke (2015) 10(3):331–5. 10.1111/ijs.12388 25338933

[30] Thomalla G Boutitie F Fiebach JB Simonsen CZ Nighoghossian N Pedraza S Stroke with unknown time of symptom onset: baseline clinical and magnetic resonance imaging data of the first thousand patients in WAKE-UP (efficacy and safety of MRI-based thrombolysis in wake-up stroke: a randomized, doubleblind, placebo-controlled trial). Stroke (2017) 48(3):770–3. 10.1161/STROKEAHA.116.015233 28174327

[31] Roveri L La Gioia S Ghidinelli C Anzalone N De Filippis C Comi G. Wake-up stroke within 3 hours of symptom awareness: imaging and clinical features compared to standard recombinant tissue plasminogen activator treated stroke. J Stroke Cerebrovasc Dis (2013) 22(6):703–8. 10.1016/j.jstrokecerebrovasdis.2011.10.003 22133742

[32] Costa R Pinho J Alves JN Amorim JM Ribeiro M Ferreira C. Wake-up stroke and stroke within the therapeutic window for thrombolysis have similar clinical severity, imaging characteristics, and outcome. J Stroke Cerebrovasc Dis (2016) 25(3):511–4. 10.1016/j.jstrokecerebrovasdis.2015.10.032 26639403

[33] Huisa BN Raman R Ernstrom K Tafreshi G Stemer A Meyer BC Alberta stroke program early CT score (ASPECTS) in patients with wake-up stroke. J Stroke Cerebrovasc Dis (2010) 19(6):475–9. 10.1016/j.jstrokecerebrovasdis.2010.03.003 20719536 PMC2974782

[34] Barber PA Demchuk AM Zhang J Buchan AM. Validity and reliability of a quantitative computed tomography score in predicting outcome of hyperacute stroke before thrombolytic therapy. ASPECTS study group. Alberta stroke programme early CT score. Lancet (2000) 355(9216):1670–4. 10.1016/S0140-6736(00)02237-6 10905241

[35] Silva GS Lima FO Camargo EC Smith WS Singhal AB Greer DM Wake-up stroke: clinical and neuroimaging characteristics. Cerebrovasc Dis (2010) 29(4):336–42. 10.1159/000278929 20130399 PMC2914433

[36] Thomalla G Rossbach P Rosenkranz M Siemonsen S Krutzelmann A Fiehler J Negative fluid-attenuated inversion recovery imaging identifies acute ischemic stroke at 3 hours or less. Ann Neurol (2009) 65(6):724–32. 10.1002/ana.21651 19557859

[37] Petkova M Rodrigo S Lamy C Oppenheim G Touze E Mas JL MR imaging helps predict time from symptom onset in patients with acute stroke: implications for patients with unknown onset time. Radiology (2010) 257(3):782–92. 10.1148/radiol.10100461 21045177

[38] Aoki J Kimura K Iguchi Y Shibazaki K Sakai K Iwanaga T. FLAIR can estimate the onset time in acute ischemic stroke patients. J Neurol Sci (2010) 293(1–2):39–44. 10.1016/j.jns.2010.03.011 20416885

[39] Ebinger M Galinovic I Rozanski M Brunecker P Endres M Fiebach JB. Fluid-attenuated inversion recovery evolution within 12 hours from stroke onset: a reliable tissue clock? Stroke (2010) 41(2):250–5. 10.1161/STROKEAHA.109.568410 20035068

[40] Legge J Graham A Male S Copeland D Lee R Goyal N Fluid-attenuated inversion recovery (FLAIR) signal intensity can identify stroke within 6 and 8 hours. J Stroke Cerebrovasc Dis (2017) 26(7):1582–7. 10.1016/j.jstrokecerebrovasdis.2017.02.030 28359617

[41] Thomalla G Cheng B Ebinger M Hao Q Tourdias T Wu O DWI-FLAIR mismatch for the identification of patients with acute ischaemic stroke within 4.5 h of symptom onset (PRE-FLAIR): a multicentre observational study. Lancet Neurol (2011) 10(11):978–86. 10.1016/S1474-4422(11)70192-2 21978972

[42] Emeriau S Serre I Toubas O Pombourcq F Oppenheim C Pierot L Can diffusion-weighted imaging-fluid-attenuated inversion recovery mismatch (positive diffusion-weighted imaging/negative fluid-attenuated inversion recovery) at 3 Tesla identify patients with stroke at <4.5 hours? Stroke (2013) 44(6):1647–51. 10.1161/STROKEAHA.113.001001 23640823

[43] Nagai K Aoki J Sakamoto Y Kimura K About 30% of wake-up stroke patients may be candidate for the tPA therapy using negative-FLAIR as a “tissue clock”. J Neurol Sci (2017) 382:101–4. 10.1016/j.jns.2017.09.042 29110999

[44] Saver JL Time is brain––quantified. Stroke (2006) 37(1):263–6. 10.1161/01.STR.0000196957.55928.ab 16339467

[45] Baird AE Warach S. Magnetic resonance imaging of acute stroke. J Cereb Blood Flow Metab (1998) 18(6):583–609. 10.1097/00004647-199806000-00001 9626183

[46] Broderick JP Palesch YY Demchuk AM Yeatts SD Khatri P Hill MD Endovascular therapy after intravenous t-PA versus t-PA alone for stroke. N Engl J Med (2013) 368(10):893–903. 10.1056/NEJMoa1214300 23390923 PMC3651875

[47] Jovin TG Chamorro A Cobo E de Miquel MA Molina CA Rovira A Thrombectomy within 8 hours after symptom onset in ischemic stroke. N Engl J Med (2015) 372(24):2296–306. 10.1056/NEJMoa1503780 25882510

[48] Goyal M Demchuk AM Menon BK Eesa M Rempel JL Thornton J Randomized assessment of rapid endovascular treatment of ischemic stroke. N Engl J Med (2015) 372(11):1019–30. 10.1056/NEJMoa1414905 25671798

[49] Campbell BC Purushotham A Christensen S Desmond PM Nagakane Y Parsons MW The infarct core is well represented by the acute diffusion lesion: sustained reversal is infrequent. J Cereb Blood Flow Metab (2012) 32(1):50–6. 10.1038/jcbfm.2011.102 21772309 PMC3323290

[50] Wintermark M Albers GW Broderick JP Demchuk AM Fiebach JB Fiehler J Acute stroke imaging research roadmap II. Stroke (2013) 44(9):2628–39. 10.1161/STROKEAHA.113.002015 23860298 PMC4040226

[51] Shih LC Saver JL Alger JR Starkman S Leary MC Vinuela F Perfusion-weighted magnetic resonance imaging thresholds identifying core, irreversibly infarcted tissue. Stroke (2003) 34(6):1425–30. 10.1161/01.STR.0000072998.70087.E9 12738899

[52] Sorensen AG Buonanno FS Gonzalez RG Schwamm LH Lev MH Huang-Hellinger FR Hyperacute stroke: evaluation with combined multisection diffusion-weighted and hemodynamically weighted echo-planar MR imaging. Radiology (1996) 199(2):391–401. 10.1148/radiology.199.2.8668784 8668784

[53] Sorensen AG Copen WA Ostergaard L Buonanno FS Gonzalez RG Rordorf G Hyperacute stroke: simultaneous measurement of relative cerebral blood volume, relative cerebral blood flow, and mean tissue transit time. Radiology (1999) 210(2):519–27. 10.1148/radiology.210.2.r99fe06519 10207439

[54] Warach S Dashe JF Edelman RR. Clinical outcome in ischemic stroke predicted by early diffusion-weighted and perfusion magnetic resonance imaging: a preliminary analysis. J Cereb Blood Flow Metab (1996) 16(1):53–9. 10.1097/00004647-199601000-00006 8530555

[55] Wu O Koroshetz WJ Ostergaard L Buonanno FS Copen WA Gonzalez RG Predicting tissue outcome in acute human cerebral ischemia using combined diffusion- and perfusion-weighted MR imaging. Stroke (2001) 32(4):933–42. 10.1161/01.STR.32.4.933 11283394

[56] Wu O Christensen S Hjort N Dijkhuizen RM Kucinski T Fiehler J Characterizing physiological heterogeneity of infarction risk in acute human ischaemic stroke using MRI. Brain (2006) 129(Pt 9):2384–93. 10.1093/brain/awl183 16891322

[57] Albers GW Thijs VN Wechsler L Kemp S Schlaug G Skalabrin E Magnetic resonance imaging profiles predict clinical response to early reperfusion: the diffusion and perfusion imaging evaluation for understanding stroke evolution (DEFUSE) study. Ann Neurol (2006) 60(5):508–17. 10.1002/ana.20976 17066483

[58] Lansberg MG Straka M Kemp S Mlynash M Wechsler LR Jovin TG MRI profile and response to endovascular reperfusion after stroke (DEFUSE 2): a prospective cohort study. Lancet Neurol (2012) 11(10):860–7. 10.1016/S1474-4422(12)70203-X 22954705 PMC4074206

[59] Fiebach JB Schellinger PD Jansen O Meyer M Wilde P Bender J CT and diffusion-weighted MR imaging in randomized order: diffusion-weighted imaging results in higher accuracy and lower interrater variability in the diagnosis of hyperacute ischemic stroke. Stroke (2002) 33(9):2206–10. 10.1161/01.STR.0000026864.20339.CB 12215588

[60] Copen WA Yoo AJ Rost NS Morais LT Schaefer PW González RG In patients with suspected acute stroke, CT perfusion-based cerebral blood flow maps cannot substitute for DWI in measuring the ischemic core. PLoS One (2017) 12(11):e0188891. 10.1371/journal.pone.0188891 29190675 PMC5708772

[61] Boned S Padroni M Rubiera M Tomasello A Coscojuela P Romero N Admission CT perfusion may overestimate initial infarct core: the ghost infarct core concept. J Neurointerv Surg (2017) 9(1):66–9. 10.1136/neurintsurg-2016-012494 27566491

[62] Lui YW Tang ER Allmendinger AM Spektor V. Evaluation of CT perfusion in the setting of cerebral ischemia: patterns and pitfalls. AJNR Am J Neuroradiol (2010) 31(9):1552–63. 10.3174/ajnr.A2026 20190208 PMC7965002

[63] Wintermark M Reichhart M Cuisenaire O Maeder P Thiran JP Schnyder P Comparison of admission perfusion computed tomography and qualitative diffusion- and perfusion-weighted magnetic resonance imaging in acute stroke patients. Stroke (2002) 33(8):2025–31. 10.1161/01.STR.0000023579.61630.AC 12154257

[64] Wintermark M Reichhart M Thiran JP Maeder P Chalaron M Schnyder P Prognostic accuracy of cerebral blood flow measurement by perfusion computed tomography, at the time of emergency room admission, in acute stroke patients. Ann Neurol (2002) 51(4):417–32. 10.1002/ana.10136 11921048

[65] Wintermark M Flanders AE Velthuis B Meuli R van Leeuwen M Goldsher D Perfusion-CT assessment of infarct core and penumbra: receiver operating characteristic curve analysis in 130 patients suspected of acute hemispheric stroke. Stroke (2006) 37(4):979–85. 10.1161/01.STR.0000209238.61459.39 16514093

[66] Abdelgawad EA Higazi MM Abdelbaky AO Abdelghany HS. Diagnostic performance of CT cerebral blood volume colour maps for evaluation of acute infarcts; comparison with diffusion-weighted MRI within 12hours of major stroke onset. J Neuroradiol (2017) 44(1):10–6. 10.1016/j.neurad.2016.10.005 27939372

[67] Campbell BC Christensen S Levi CR Desmond PM Donnan GA Davis SM Cerebral blood flow is the optimal CT perfusion parameter for assessing infarct core. Stroke (2011) 42(12):3435–40. 10.1161/STROKEAHA.111.618355 21980202

[68] Deipolyi AR Wu O Macklin EA Schaefer PW Schwamm LH Gilberto Gonzalez R Reliability of cerebral blood volume maps as a substitute for diffusion-weighted imaging in acute ischemic stroke. J Magn Reson Imaging (2012) 36(5):1083–7. 10.1002/jmri.23740 22761110 PMC4110961

[69] Geuskens RR Borst J Lucas M Boers AM Berkhemer OA Roos YB Characteristics of misclassified CT perfusion ischemic core in patients with acute ischemic stroke. PLoS One (2015) 10(11):e0141571. 10.1371/journal.pone.0141571 26536226 PMC4633055

[70] Schaefer PW Souza L Kamalian S Hirsch JA Yoo AJ Gonzalez RG Limited reliability of computed tomographic perfusion acute infarct volume measurements compared with diffusion-weighted imaging in anterior circulation stroke. Stroke (2015) 46(2):419–24. 10.1161/STROKEAHA.114.007117 25550366 PMC4308477

[71] Copen WA Morais LT Wu O Schwamm LH Schaefer PW González RG In acute stroke, can CT perfusion-derived cerebral blood volume maps substitute for diffusion-weighted imaging in identifying the ischemic core? PLoS One (2015) 10(7):e0133566. 10.1371/journal.pone.0133566 26193486 PMC4508041

[72] Mishra NK Christensen S Wouters A Campbell BC Straka M Mlynash M Reperfusion of very low cerebral blood volume lesion predicts parenchymal hematoma after endovascular therapy. Stroke (2015) 46(5):1245–9. 10.1161/STROKEAHA.114.008171 25828235 PMC4414872

[73] Alsop DC Makovetskaya E Kumar S Selim M Schlaug G. Markedly reduced apparent blood volume on bolus contrast magnetic resonance imaging as a predictor of hemorrhage after thrombolytic therapy for acute ischemic stroke. Stroke (2005) 36(4):746–50. 10.1161/01.STR.0000158913.91058.93 15746457

[74] Hermitte L Cho TH Ozenne B Nighoghossian N Mikkelsen IK Ribe L Very low cerebral blood volume predicts parenchymal hematoma in acute ischemic stroke. Stroke (2013) 44(8):2318–20. 10.1161/STROKEAHA.113.001751 23723309

[75] Campbell BC Christensen S Butcher KS Gordon I Parsons MW Desmond PM Regional very low cerebral blood volume predicts hemorrhagic transformation better than diffusion-weighted imaging volume and thresholded apparent diffusion coefficient in acute ischemic stroke. Stroke (2010) 41(1):82–8. 10.1161/STROKEAHA.109.562116 19959537

[76] Protto S Pienimäki JP Seppänen J Numminen H Sillanpää N. Low cerebral blood volume identifies poor outcome in stent retriever thrombectomy. Cardiovasc Intervent Radiol (2017) 40(4):502–9. 10.1007/s00270-016-1532-x 27942925

[77] Tsogkas I Knauth M Schregel K Behme D Wasser K Maier I Added value of CT perfusion compared to CT angiography in predicting clinical outcomes of stroke patients treated with mechanical thrombectomy. Eur Radiol (2016) 26(11):4213–9. 10.1007/s00330-016-4257-y 26905866

[78] Hacke W Albers G Al-Rawi Y Bogousslavsky J Davalos A Eliasziw M The Desmoteplase in Acute Ischemic Stroke Trial (DIAS): a phase II MRI-based 9-hour window acute stroke thrombolysis trial with intravenous desmoteplase. Stroke (2005) 36(1):66–73. 10.1161/01.STR.0000149938.08731.2c 15569863

[79] Hacke W Furlan AJ Al-Rawi Y Davalos A Fiebach JB Gruber F Intravenous desmoteplase in patients with acute ischaemic stroke selected by MRI perfusion-diffusion weighted imaging or perfusion CT (DIAS-2): a prospective, randomised, double-blind, placebo-controlled study. Lancet Neurol (2009) 8(2):141–50. 10.1016/S1474-4422(08)70267-9 19097942 PMC2730486

[80] Furlan AJ Eyding D Albers GW Al-Rawi Y Lees KR Rowley HA Dose escalation of Desmoteplase for Acute Ischemic Stroke (DEDAS): evidence of safety and efficacy 3 to 9 hours after stroke onset. Stroke (2006) 37(5):1227–31. 10.1161/01.STR.0000217403.66996.6d 16574922

[81] Fiebach JB Al-Rawi Y Wintermark M Furlan AJ Rowley HA Lindstén A Vascular occlusion enables selecting acute ischemic stroke patients for treatment with desmoteplase. Stroke (2012) 43(6):1561–6. 10.1161/STROKEAHA.111.642322 22474060

[82] Albers GW von Kummer R Truelsen T Jensen JK Ravn GM Grønning BA Safety and efficacy of desmoteplase given 3-9 h after ischaemic stroke in patients with occlusion or high-grade stenosis in major cerebral arteries (DIAS-3): a double-blind, randomised, placebo-controlled phase 3 trial. Lancet Neurol (2015) 14(6):575–84. 10.1016/S1474-4422(15)00047-2 25937443

[83] von Kummer R Mori E Truelsen T Jensen JS Grønning BA Fiebach JB Desmoteplase 3 to 9 hours after major artery occlusion stroke: the DIAS-4 trial (efficacy and safety study of desmoteplase to treat acute ischemic stroke). Stroke (2016) 47(12):2880–7. 10.1161/STROKEAHA.116.013715 27803391

[84] Davis SM Donnan GA Parsons MW Levi C Butcher KS Peeters A Effects of alteplase beyond 3 h after stroke in the Echoplanar Imaging Thrombolytic Evaluation trial (EPITHET): a placebo-controlled randomised trial. Lancet Neurol (2008) 7(4):299–309. 10.1016/S1474-4422(08)70044-9 18296121

[85] Nagakane Y Christensen S Brekenfeld C Ma H Churilov L Parsons MW EPITHET: positive result after reanalysis using baseline diffusion-weighted imaging/perfusion-weighted imaging co-registration. Stroke (2011) 42(1):59–64. 10.1161/STROKEAHA.110.580464 21127303

[86] Parsons M Spratt N Bivard A Campbell B Chung K Miteff F A randomized trial of tenecteplase versus alteplase for acute ischemic stroke. N Engl J Med (2012) 366(12):1099–107. 10.1056/NEJMoa1109842 22435369

[87] Logallo N Novotny V Assmus J Kvistad CE Alteheld L Ronning OM Tenecteplase versus alteplase for management of acute ischaemic stroke (NOR-TEST): a phase 3, randomised, open-label, blinded endpoint trial. Lancet Neurol (2017) 16(10):781–8. 10.1016/S1474-4422(17)30253-3 28780236

[88] Tenecteplase in Wake-Up Ischaemic Stroke Trial. Available from: https://ClinicalTrials.gov/show/NCT03181360 (Accessed: February 15, 2018).

[89] Kidwell CS Jahan R Gornbein J Alger JR Nenov V Ajani Z A trial of imaging selection and endovascular treatment for ischemic stroke. N Engl J Med (2013) 368(10):914–23. 10.1056/NEJMoa1212793 23394476 PMC3690785

[90] Kidwell CS Wintermark M De Silva DA Schaewe TJ Jahan R Starkman S Multiparametric MRI and CT models of infarct core and favorable penumbral imaging patterns in acute ischemic stroke. Stroke (2013) 44(1):73–9. 10.1161/STROKEAHA.112.670034 23233383 PMC3558033

[91] Dávalos A Blanco M Pedraza S Leira R Castellanos M Pumar JM The clinical-DWI mismatch: a new diagnostic approach to the brain tissue at risk of infarction. Neurology (2004) 62(12):2187–92. 10.1212/01.WNL.0000130570.41127.EA 15210880

[92] Tei H Uchiyama S Usui T. Clinical-diffusion mismatch defined by NIHSS and ASPECTS in non-lacunar anterior circulation infarction. J Neurol (2007) 254(3):340–6. 10.1007/s00415-006-0368-8 17345045

[93] Prosser J Butcher K Allport L Parsons M MacGregor L Desmond P Clinical-diffusion mismatch predicts the putative penumbra with high specificity. Stroke (2005) 36(8):1700–4. 10.1161/01.STR.0000173407.40773.17 16020762

[94] Lansberg MG Thijs VN Hamilton S Schlaug G Bammer R Kemp S Evaluation of the clinical-diffusion and perfusion-diffusion mismatch models in DEFUSE. Stroke (2007) 38(6):1826–30. 10.1161/STROKEAHA.106.480145 17495217 PMC3985733

[95] Ebinger M Iwanaga T Prosser JF De Silva DA Christensen S Collins M Clinical-diffusion mismatch and benefit from thrombolysis 3 to 6 hours after acute stroke. Stroke (2009) 40(7):2572–4. 10.1161/STROKEAHA.109.548073 19407226

[96] Nogueira RG Kemmling A Souza LM Payabvash S Hirsch JA Yoo AJ Clinical diffusion mismatch better discriminates infarct growth than mean transit time-diffusion weighted imaging mismatch in patients with middle cerebral artery-M1 occlusion and limited infarct core. J Neurointerv Surg (2017) 9(2):127–30. 10.1136/neurintsurg-2014-011602 26957483

[97] Messé SR Kasner SE Chalela JA Cucchiara B Demchuk AM Hill MD CT-NIHSS mismatch does not correlate with MRI diffusion-perfusion mismatch. Stroke (2007) 38(7):2079–84. 10.1161/STROKEAHA.106.480731 17540971

[98] Kent DM Hill MD Ruthazer R Coutts SB Demchuk AM Dzialowski I “Clinical-CT mismatch” and the response to systemic thrombolytic therapy in acute ischemic stroke. Stroke (2005) 36(8):1695–9. 10.1161/01.STR.0000173397.31469.4b 16002756

[99] Boxerman JL Jayaraman MV Mehan WA Rogg JM Haas RA. Clinical stroke penumbra: use of National Institutes Of Health Stroke Scale as a surrogate for CT perfusion in patient triage for intra-arterial middle cerebral artery stroke therapy. AJNR Am J Neuroradiol (2012) 33(10):1893–900. 10.3174/ajnr.A3102 22627795 PMC7964623

[100] Souza LC Yoo AJ Chaudhry ZA Payabvash S Kemmling A Schaefer PW Malignant CTA collateral profile is highly specific for large admission DWI infarct core and poor outcome in acute stroke. AJNR Am J Neuroradiol (2012) 33(7):1331–6. 10.3174/ajnr.A2985 22383238 PMC3888794

[101] Vagal A Menon BK Foster LD Livorine A Yeatts SD Qazi E Association between CT angiogram collaterals and CT perfusion in the interventional management of stroke III trial. Stroke (2016) 47(2):535–8. 10.1161/STROKEAHA.115.011461 26658448 PMC4729636

[102] Liebeskind DS Tomsick TA Foster LD Yeatts SD Carrozzella J Demchuk AM Collaterals at angiography and outcomes in the interventional management of stroke (IMS) III trial. Stroke (2014) 45(3):759–64. 10.1161/STROKEAHA.113.004072 24473178 PMC3977615

[103] Marks MP Lansberg MG Mlynash M Olivot JM Straka M Kemp S Effect of collateral blood flow on patients undergoing endovascular therapy for acute ischemic stroke. Stroke (2014) 45(4):1035–9. 10.1161/STROKEAHA.113.004085 24569816 PMC4396867

[104] Kawano H Bivard A Lin L Spratt NJ Miteff F Parsons MW Relationship between collateral status, contrast transit, and contrast density in acute ischemic stroke. Stroke (2016) 47(3):742–9. 10.1161/STROKEAHA.115.011320 26839354

[105] Cho AH Sohn SI Han MK Lee DH Kim JS Choi CG Safety and efficacy of MRI-based thrombolysis in unclear-onset stroke. A preliminary report. Cerebrovasc Dis (2008) 25(6):572–9. 10.1159/000132204 18483457

[106] Barreto AD Martin-Schild S Hallevi H Morales MM Abraham AT Gonzales NR Thrombolytic therapy for patients who wake-up with stroke. Stroke (2009) 40(3):827–32. 10.1161/STROKEAHA.108.528034 19131657 PMC2676861

[107] Manawadu D Bodla S Jarosz J Keep J Kalra L. A case-controlled comparison of thrombolysis outcomes between wake-up and known time of onset ischemic stroke patients. Stroke (2013) 44(8):2226–31. 10.1161/STROKEAHA.112.673145 23723307

[108] Natarajan SK Karmon Y Snyder KV Ohta H Hauck EF Hopkins LN Prospective acute ischemic stroke outcomes after endovascular therapy: a real-world experience. World Neurosurg (2010) 74(4–5):455–64. 10.1016/j.wneu.2010.06.035 21492595

[109] Aghaebrahim A Leiva-Salinas C Jadhav AP Jankowitz B Zaidi S Jumaa M Outcomes after endovascular treatment for anterior circulation stroke presenting as wake-up strokes are not different than those with witnessed onset beyond 8 hours. J Neurointerv Surg (2015) 7(12):875–80. 10.1136/neurintsurg-2014-011316 25326003

[110] Jovin TG Liebeskind DS Gupta R Rymer M Rai A Zaidat OO Imaging-based endovascular therapy for acute ischemic stroke due to proximal intracranial anterior circulation occlusion treated beyond 8 hours from time last seen well: retrospective multicenter analysis of 237 consecutive patients. Stroke (2011) 42(8):2206–11. 10.1161/STROKEAHA.110.604223 21778444

[111] Ribo M Flores A Rubiera M Pagola J Sargento-Freitas J Rodriguez-Luna D Extending the time window for endovascular procedures according to collateral pial circulation. Stroke (2011) 42(12):3465–9. 10.1161/STROKEAHA.111.623827 21960574

[112] Hwang YH Kang DH Kim YW Kim YS Park SP Liebeskind DS. Impact of time-to-reperfusion on outcome in patients with poor collaterals. AJNR Am J Neuroradiol (2015) 36(3):495–500. 10.3174/ajnr.A4151 25376808 PMC8013039

[113] Adams HP Jr Effron MB Torner J Davalos A Frayne J Teal P Emergency administration of abciximab for treatment of patients with acute ischemic stroke: results of an international phase III trial: abciximab in emergency treatment of stroke trial (AbESTT-II). Stroke (2008) 39(1):87–99. 10.1161/STROKEAHA.106.476648 18032739

[114] Adams HP Jr Leira EC Torner JC Barnathan E Padgett L Effron MB Treating patients with ‘wake-up’ stroke: the experience of the AbESTT-II trial. Stroke (2008) 39(12):3277–82. 10.1161/STROKEAHA.107.508853 18772451

[115] Barreto AD Fanale CV Alexandrov AV Gaffney KC Vahidy FS Nguyen CB Prospective, open-label safety study of intravenous recombinant tissue plasminogen activator in wake-up stroke. Ann Neurol (2016) 80(2):211–8. 10.1002/ana.24700 27273860

[116] ClinicalTrials.gov Safety of Intravenous Thrombolytics in Stroke on Awakening. Available from: https://ClinicalTrials.gov/show/NCT01643902 (Accessed: February 15, 2018).

[117] Kang DW Sohn SI Hong KS Yu KH Hwang YH Han MK Reperfusion therapy in unclear-onset stroke based on MRI evaluation (RESTORE): a prospective multicenter study. Stroke (2012) 43(12):3278–83. 10.1161/STROKEAHA.112.675926 23093613

[118] Aoki J Kimura K Iguchi Y Shibazaki K Iwanaga T Watanabe M Intravenous thrombolysis based on diffusion-weighted imaging and fluid-attenuated inversion recovery mismatch in acute stroke patients with unknown onset time. Cerebrovasc Dis (2011) 31(5):435–41. 10.1159/000323850 21346348

[119] Schwamm LH Wu O Song SS Latour LL Ford AL Hsia A Intravenous thrombolysis in unwitnessed stroke onset: MR WITNESS trial results. Ann Neurol (2018). 10.1002/ana.25235 PMC6095471 29689135

[120] Koga M Toyoda K Kimura K Yamamoto H Sasaki M Hamasaki T THrombolysis for acute wake-up and unclear-onset strokes with alteplase at 0.6 mg/kg (THAWS) trial. Int J Stroke (2014) 9(8):1117–24. 10.1111/ijs.12360 25088843 PMC4660886

[121] Ma H Parsons MW Christensen S Campbell BC Churilov L Connelly A A multicentre, randomized, double-blinded, placebo-controlled Phase III study to investigate extending the time for thrombolysis in emergency neurological deficits (EXTEND). Int J Stroke (2012) 7(1):74–80. 10.1111/j.1747-4949.2011.00730.x 22188854

[122] ClinicalTrials.gov Efficacy and Safety of Thrombectomy in Stroke With Extended Lesion and Extended Time Window. Available from: https://ClinicalTrials.gov/show/NCT03094715 (Accessed: February 15, 2018). 10.1177/1747493018798558 PMC6604397 30156479

